# Functional Constraints on Replacing an Essential Gene with Its Ancient and Modern Homologs

**DOI:** 10.1128/mBio.01276-17

**Published:** 2017-08-29

**Authors:** Betül Kacar, Eva Garmendia, Nurcan Tuncbag, Dan I. Andersson, Diarmaid Hughes

**Affiliations:** a Department of Organismic and Evolutionary Biology, Harvard University, Cambridge, Massachusetts, USA; b Department of Medical Biochemistry and Microbiology, Uppsala University, Uppsala, Sweden; c Department of Health Informatics, Graduate School of Informatics, METU, Ankara, Turkey; d Cancer Systems Biology Laboratory (CanSyL), METU, Ankara, Turkey; Massachusetts Institute of Technology

**Keywords:** EF-Tu, horizontal gene transfer, ancient genes, proteobacteria, *tuf*

## Abstract

Genes encoding proteins that carry out essential informational tasks in the cell, in particular where multiple interaction partners are involved, are less likely to be transferable to a foreign organism. Here, we investigated the constraints on transfer of a gene encoding a highly conserved informational protein, translation elongation factor Tu (EF-Tu), by systematically replacing the endogenous *tufA* gene in the *Escherichia coli* genome with its extant and ancestral homologs. The extant homologs represented *tuf* variants from both near and distant homologous organisms. The ancestral homologs represented phylogenetically resurrected *tuf* sequences dating from 0.7 to 3.6 billion years ago (bya). Our results demonstrate that all of the foreign *tuf* genes are transferable to the *E. coli* genome, provided that an additional copy of the EF-Tu gene, *tufB*, remains present in the *E. coli* genome. However, when the *tufB* gene was removed, only the variants obtained from the gammaproteobacterial family (extant and ancestral) supported growth which demonstrates the limited functional interchangeability of *E. coli tuf* with its homologs. Relative bacterial fitness correlated with the evolutionary distance of the extant *tuf* homologs inserted into the *E. coli* genome. This reduced fitness was associated with reduced levels of EF-Tu and reduced rates of protein synthesis. Increasing the expression of *tuf* partially ameliorated these fitness costs. In summary, our analysis suggests that the functional conservation of protein activity, the amount of protein expressed, and its network connectivity act to constrain the successful transfer of this essential gene into foreign bacteria.

## IMPORTANCE

Horizontal gene transfer (HGT) is a fundamental driving force in bacterial evolution. However, whether essential genes can be acquired by HGT and whether they can be acquired from distant organisms are very poorly understood. By systematically replacing *tuf* with ancestral homologs and homologs from distantly related organisms, we investigated the constraints on HGT of a highly conserved gene with multiple interaction partners. The ancestral homologs represented phylogenetically resurrected *tuf* sequences dating from 0.7 to 3.6 bya. Only variants obtained from the gammaproteobacterial family (extant and ancestral) supported growth, demonstrating the limited functional interchangeability of *E. coli tuf* with its homologs. Our analysis suggests that the functional conservation of protein activity, the amount of protein expressed, and its network connectivity act to constrain the successful transfer of this essential gene into foreign bacteria.

## INTRODUCTION

The complexity hypothesis assigns the function and the network interactivity of a gene product as two primary factors determining a gene’s capacity to successfully transfer and adapt in another genome (1–5). Consequently, genes involved in central informational tasks (i.e., replication, transcription, and translation) are expected to be less transferable to a foreign genome than genes involved in other activities (1, 6, 7).

Genomic replacement of an essential gene with another homolog may potentially disturb the gene product’s function depending on the degree of functional equivalence or compatibility between the two genes (8–11). Alterations in gene dosage resulting from the acquisition of a foreign gene may also perturb cellular homeostasis, resulting in a lower transcription rate or an altered global pattern of gene expression, network function, and organismal survivability (11–18).

Genes involved in the translation of mRNA into protein perform one of the most crucial informational tasks in the cell, and based on phylogenomic analysis, they are expected to be highly resistant to gene transfer (1, 19). There are examples demonstrating that some ribosomal protein genes can be integrated into foreign genomes under certain conditions (20–23). However, no study has systematically tested whether there is a direct correlation between organism fitness and the evolutionary distance between an essential endogenous gene and its substituted (ancestral or extant) homolog.

Here, we focus on the bacterial elongation factor Tu (EF-Tu) protein, encoded by *tuf*, one of the most ancient and highly conserved proteins. EF-Tu has an essential function in the translation machinery by delivering aminoacylated tRNA (aa-tRNA) molecules into the A site of the ribosome (24). EF-Tu is encoded by two genes in *Escherichia coli*, *tufA* and *tufB*, generated by an ancient duplication event thought to be specific to the proteobacterial lineage preceding the Cambrian period (25, 26). Expression of EF-Tu is primarily driven by *tufA*, with 66% of cellular EF-Tu expressed from the *tufA* gene (27). EF-Tu protein levels in the cell are correlated with cellular fitness and intrinsically regulated in order to maintain growth rate (28–30). EF-Tu belongs to the ancient protein repertoire of the cell, evolves slowly, and serves as a functional fossil by participating in ancient and conserved functions (31). It remains unclear whether *tuf* genes are replaceable by their ancient counterparts or homologs obtained from an extant organism. Answering this question would allow us to explore the limits of interchangeability for the *E. coli tuf* gene and to ascertain a pattern within and between bacterial lineages across time and divergence. We sought guidance from a methodology referred to as ancestral sequence reconstruction (32–37) and accessed reconstructed ancestral *tuf* variants (35) as well as modern *tuf* gene sequences in order to observe the patterns of interchangeability among multiple nodes along the EF-Tu phylogenetic tree. We utilized a set of foreign genes representing *E. coli* EF-Tu homologs from closely and distantly related bacteria, as well as phylogenetically inferred ancestral EF-Tu proteins dating from 0.7 to 3.6 billion years ago (bya), thus accessing interspecies (modern) and ancestral (paleogenetic) axes. We determined the fitness effects of the introduction of a foreign *tuf* gene into each strain by replacing the native *E. coli tufA* gene with foreign variants and asked whether these foreign genes could support cell viability when the *tufB* gene was removed from the chromosome. We examined the impact of the *tuf* gene replacements on growth rate and protein levels, as well as on protein function-structure, and assessed the extent of lateral and ancestral phylogenetic distances between the alien gene and host genome that yielded viable organisms.

## RESULTS

### Replacement of the *tufA* gene in *E. coli* reduces relative fitness.

**FIG 1:**
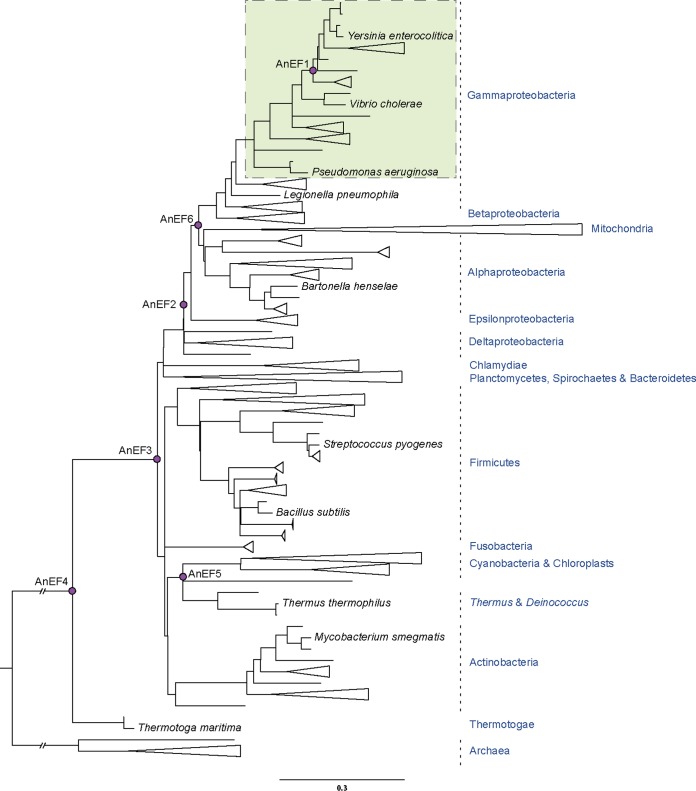
Phylogenetic tree indicating the node and taxa of the ancestral and modern *tuf* (EF-Tu) homologs. Pink circles represent the ancestral EF-Tu nodes. *E. coli* was genetically engineered to carry ancestral or modern homologs of *tuf*, encoding translation elongation factor EF-Tu, replacing the native *E. coli tufA* gene. A shaded box indicates the area of viability of EF-Tu gene exchange. The scale bar expresses units of amino acid substitutions per site. The tree was created with data from references 35 and 38.

Using genetic recombineering, we generated a set of *E. coli* strains in which the *tufA* gene coding sequence was precisely replaced by the coding sequence of its ancestral and modern homologs (Fig. 1; see also Fig. S1 in the supplemental material). The 16 *tuf* homologs cover bacterial species from a wide span of taxa (*Yersinia enterocolitica*, *Vibrio cholerae*, *Pseudomonas aeruginosa*, *Legionella pneumophila*, *Bartonella henselae*, *Streptococcus pyogenes*, *Bacillus subtilis*, *Thermus thermophilus*, *Mycobacterium smegmatis*, and *Thermotoga maritima*) as well as six ancestral sequences that extend deep into the bacterial phylogenetic tree (Fig. 1). These six sequences represent ancestral nodes dating from approximately 0.7 bya back to the last common ancestor of bacterial *tuf*, dated to approximately 3.6 bya (35, 38). These homologs of *tuf* encode EF-Tu variants that range in amino acid identity from 93.9% (*Y. enterocolitica*) to 69.4% (*T. maritima*) relative to *E. coli* EF-Tu (Fig. S2). Nucleotide sequences of the ancestral *tuf* genes are shown in Table S1.

10.1128/mBio.01276-17.1 FIG S1  Foreign *tuf* genes were integrated into the bacterial genome in a stepwise fashion as shown. (A) Each of the foreign *tuf* genes was integrated into the *E. coli* chromosome (replacing native *tufA* from start to stop codons). (B) In each strain carrying a foreign *tuf* gene at the *tufA* locus, an attempt was made to remove the native *E. coli tufB* gene (replacing the coding sequence from start to stop codons). Download FIG S1, PDF file, 0.2 MB. Copyright © 2017 Kacar et al. 2017 Kacar et al. This content is distributed under the terms of the Creative Commons Attribution 4.0 International license.

10.1128/mBio.01276-17.2 FIG S2  Relative fitness of strains carrying two *tuf* genes (*tufA*-foreign and *tufB E. coli*) as a function of percent identity to *E. coli* EF-Tu (indicated as 100%). Strains shown in green carry a foreign *tuf* gene that can support viability when the *E. coli tufB* gene has been deleted. Download FIG S2, PDF file, 0.2 MB. Copyright © 2017 Kacar et al. 2017 Kacar et al. This content is distributed under the terms of the Creative Commons Attribution 4.0 International license.

10.1128/mBio.01276-17.6 TABLE S1  Nucleotide sequences of ancestral *tuf* genes. Download TABLE S1, PDF file, 0.1 MB. Copyright © 2017 Kacar et al. 2017 Kacar et al. This content is distributed under the terms of the Creative Commons Attribution 4.0 International license.

**FIG 2:**
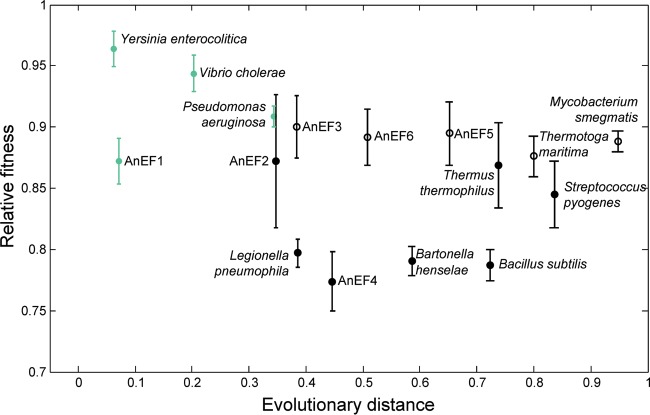
Correlation between relative fitness and evolutionary distance for bacterial strains carrying a foreign *tuf* gene. Relative fitness is shown as a function of evolutionary divergence (see Materials and Methods) of EF-Tu homologs from *E. coli* (1 indicates greatest difference from *E. coli*). Each *E. coli* strain carried a foreign *tuf* gene at the *tufA* location and an intact native *tufB* gene. Fitness was measured as exponential growth rate, relative to the *E. coli* wild type carrying *tufA* and *tufB*. Species names and ancestor notations (AnEF, ancestral EF-Tu) refer to the source of the foreign *tuf* gene sequence in each strain. Strains shown in green carry a foreign *tuf* gene that can support viability even when the *E. coli tufB* gene has been deleted. Strains shown in black carry foreign *tuf* genes that do not support viability in the absence of the *E. coli tufB* gene. Empty symbols represent strains where the *tufB* region was amplified.

To construct a set of isogenic strains, the endogenous *E. coli tufA* gene was replaced with each of the *tuf* variants while the second endogenous *tuf* gene, *tufB*, remained intact in the genome. All engineered strains in which *tufA* was replaced with a foreign *tuf* gene retained viability (Fig. 2). The effects on relative fitness of the foreign *tuf* genes were determined by measuring the exponential growth rates in LB and relating them to that of the isogenic wild type (carrying native *tufA* and *tufB*), where relative fitness was set to 1.0. The relative fitness of each of the engineered constructs varied from 0.96 down to 0.77 (Fig. 2). The relative fitness of *E. coli* in which *tufA* was deleted from the chromosome was 0.7. The similarity in relative fitness between *E. coli* lacking *tufA* and some of the strains carrying foreign *tuf* genes raised the question of whether all of the foreign genes would be capable of supporting viability in the absence of a functioning *tufB* gene.

Because the *tufB* gene is located between the rRNA operons *rrnB* and *rrnE*, in a region that is subject to frequent amplification (39), we asked whether *tufB* was amplified in any of the strains carrying a foreign *tuf* allele. Our expectation was that a less effective, or completely inactive, foreign *tuf* gene might select for genomes in which *tufB* was amplified as a fitness-compensatory mechanism. Using real-time quantitative PCR (RT-qPCR), we found that the *tufB* region was duplicated or triplicated in 5 of the 16 engineered strains (Fig. 2). The 5 strains in which the *tufB* region was amplified are those that carry the most distant *tuf* homologs, compatible with selection for improved fitness.

### A subset of foreign *tuf* genes supports viability.

We next asked whether any of the foreign *tuf* genes could support viability in the absence of *E. coli tufB*, by attempting to remove the endogenous *tufB* gene from the chromosome of each of the 16 strains carrying a foreign *tuf* gene, as outlined in Fig. S1. In the absence of *tufB*, the only foreign *tuf* sequences that supported viability were those from *Y. enterocolitica*, *V. cholerae*, and *P. aeruginosa* and AnEF1, the youngest of the ancestral *tuf* genes at ca. 0.7 bya (Fig. 2), whereas the phylogenetically more distant *tuf* genes did not. We were interested to know how strains carrying the different viable *tuf* homologs were affected in each phase of the growth cycle. To do this, we measured length of lag phase, doubling time (DT) in exponential growth phase, and final optical density (OD) achieved in stationary phase. The parameters differed significantly between LB and DM25 (Davis minimal medium with 25 mg/liter glucose), with shorter lag times, shorter doubling times, and higher final ODs associated with growth in LB for each of the homologs (Table 1). The ranking of the parameters differed somewhat between LB and DM25. In rich medium, where growth rates are highest, the translation system, including EF-Tu, represents a major fraction of the bacterial mass, arguing that under these conditions any maladaptations will be more likely to reduce physiological fitness. In agreement, the strain carrying the native *E. coli tuf* gene had the shortest doubling time, shortest lag time, and maximum final OD in LB relative to strains carrying any of the other *tuf* homologs (Table 1). In the remainder of the text, we use doubling time in LB as a proxy for relative fitness. One striking indication that foreign *tuf* homologs can be maladapted is seen for *tuf* from *V. cholerae*, where the lag time in LB is 30- to 40-fold longer than that associated with any of the other *tuf* genes.

**TABLE 1:** Growth characteristics of strains harboring only one *tuf* gene

*tuf* gene	Growth in medium, mean ± SD^a^					
	LB			DM25		
	Lag time (min)	Doubling time (min)	Max OD_600_	Lag time (min)	Doubling time (min)	Max OD_600_
*Escherichia coli*	6.8 ± 0.8	25.0 ± 0.3	1.39 ± 0.02	124.0 ± 3.0	81.7 ± 3.3	0.60 ± 0.01
*Yersinia enterocolitica*	8.2 ± 1.2	26.3 ± 0.4	1.39 ± 0.02	133.0 ± 8.6	80.0 ± 1.4	0.61 ± 0.01
*Vibrio cholerae*	300.2 ± 11.5	41.5 ± 2.2	1.38 ± 0.02	745.4 ± 36.1	134.5 ± 15.3	0.58 ± 0.05
AnEF1	10.8 ± 1.5	48.4 ± 0.9	1.23 ± 0.02	73.1 ± 4.1	88.9 ± 3.2	0.53 ± 0.01
*Pseudomonas aeruginosa*	7.3 ± 5.5	66.9 ± 2.6	1.24 ± 0.02	87.7 ± 6.7	134.1 ± 3.0	0.63 ± 0.02

a LB, Luria-Bertani broth; DM25, Davis minimal medium (25 mg/liter glucose). Each value represents the mean from 3 biological replicates.

### Fitness correlates with EF-Tu protein levels and rate of protein synthesis.

**FIG 3:**
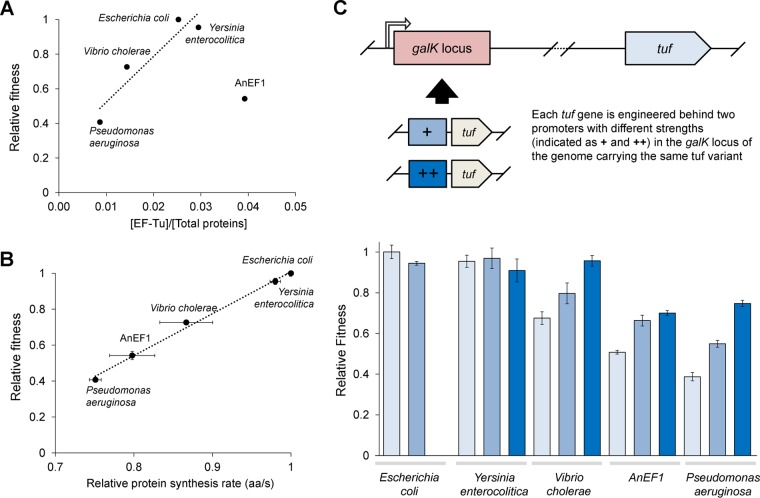
Fitness characteristics of strains carrying single *tuf* genes. (A) Relative fitness of strains carrying a single *tuf* gene, at the *tufA* locus, as a function of EF-Tu protein produced. EF-Tu concentration was normalized to the total protein concentration. Species names indicate the species origin of the only *tuf* gene present (linear regression involves only extant sequences; *R*^2^ = 0.938). (B) Relative protein synthesis rate (amino acids/second) of the ribosome in each constructed bacterial strain (*R*^2^ = 0.994), determined by the time that it takes to produce β-galactosidase activity. Relative fitness of strains carrying a single *tuf* gene at the *tufA* locus correlates with the rate of protein synthesis. Results are means for three biological replicates with error bars representing standard deviation values. (C) Relative fitness of strains expressing each of the five *tuf* genes at three different levels: no additional *tuf* (light blue), additional *tuf* expression under promoter J23105 (medium blue), and additional *tuf* expression under promoter J23100 (dark blue). Promoter J23105 expresses the gene at a medium level (represented by +), whereas promoter J23100 expresses the gene at a higher level (represented by ++). Results are means for three biological replicates with error bars representing standard deviation values.

A key question is why the viable EF-Tu gene replacements reduce fitness. Two possibilities (which are not mutually exclusive) are that the foreign genes are suboptimally expressed (i.e., a concentration problem) and that they are suboptimal in their function and interaction with the protein synthesis machinery and cellular network (i.e., an activity or toxicity problem). To evaluate the possibility that the foreign genes were suboptimally expressed, we measured EF-Tu abundance by liquid chromatography-tandem mass spectrometry (LC-MS/MS) in each of the viable strains (Fig. 3A). This analysis demonstrated that the level of EF-Tu relative to total protein varied significantly between engineered strains carrying different foreign *tuf* genes (Fig. 3A). With the exception of the ancestral gene, AnEF1, there is a good correlation between relative fitness of the extant species’ genes and the concentration of EF-Tu (*R*^2^ value, 0.938). The mechanism resulting in these concentration differences is not known, but neither fitness nor concentration correlates with differences in codon usage between the *tuf* genes (Fig. S3). The proteomics data suggest that in most cases at least some of the reduction in relative fitness associated with foreign *tuf* genes is because bacteria do not produce an adequate level of EF-Tu to support fast growth. In the case of AnEF1, the reduced fitness may be more closely associated with reduced specific activity and/or toxicity.

10.1128/mBio.01276-17.3 FIG S3  Codon adaptation index analysis. Species names indicate the species origin of the only *tuf* gene present. (A) Codon adaptation index as a function of the relative fitness of the strains. (B) Codon adaptation index as a function of the EF-Tu concentration normalized to the total protein content. Codon adaptation index was calculated with the CAI Calculator 2 using the Sharp and Li equation (P. M. Sharp and W.-H. Li, Nucleic Acids Res 15:1281–1295, 1987). Download FIG S3, PDF file, 0.3 MB. Copyright © 2017 Kacar et al. 2017 Kacar et al. This content is distributed under the terms of the Creative Commons Attribution 4.0 International license.

As an additional assay of the suboptimal functionality of foreign *tuf* genes as a possible reason for reduced fitness, we asked whether they supported a similar rate of protein synthesis as *E. coli tufA*. We constructed a set of isogenic strains suitable for measuring protein synthesis step time, using β-galactosidase as a model protein. Step time varied between the 5 different *tuf* genes, with the highest rate associated with *tuf* from *E. coli* and the lowest rate associated with *tuf* from *P. aeruginosa* (Fig. 3B). The data showed a very good correlation between protein synthesis step time and relative fitness (*R*^2^ value, 0.994).

Given the observed correlation between EF-Tu amount and relative fitness, we asked whether increasing the amount of EF-Tu would ameliorate the fitness costs. To test this, we engineered strains carrying these five *tuf* genes, placing an additional copy of each gene into the chromosome under the control of either of two different constitutive promoters, J23105 and J23100 (where J23100 is the stronger promoter [see Materials and Methods]). This created a set of isogenic strains putatively expressing each of the five *tuf* genes at three different levels. The exponential growth rate in LB was measured for each strain (Fig. 3C). For the three *tuf* genes associated with significantly reduced fitness relative to *E. coli tufA* (*V. cholerae*, AnEF1, and *P. aeruginosa*), overexpression was associated with a significant reduction in doubling time (higher growth rate) and in the case of *V. cholerae* reached a growth rate almost as high as that supported by the single copy of *tufA* in *E. coli*. In contrast, overexpression of *Y. enterocolitica tuf*, where a single copy already supported a growth rate close to that of *E. coli tufA*, did not further increase the growth rate (Fig. 3C). These data support the conclusion that an effectively low level of EF-Tu is one cause of the reduced fitness associated with foreign *tuf* genes.

Overall, these experiments show that the differences in relative fitness cost associated with different foreign *tuf* genes in *E. coli* correlate both with the amount of EF-Tu protein (measured by MS and overproduced from additional genes) and with the relative rate of protein synthesis supported by each *tuf* gene.

### Conservation of EF-Tu residues correlates with viability in *E. coli.*

**FIG 4:**
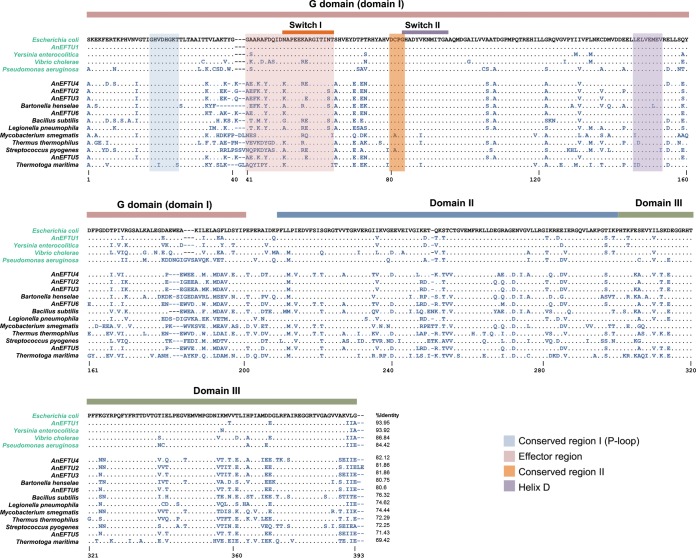
Alignment of EF-Tu protein sequences encoded by foreign *tuf* variants. EF-Tu sequences were aligned using Clustal Omega. Sequences labeled in green indicate that the foreign *tuf* gene supports viability as the only *tuf* gene in the genome. Sequences labeled in black indicate that the strain requires *E. coli tufB* for viability.

The established function of EF-Tu is to deliver aminoacylated tRNAs into the A site on mRNA-programmed ribosomes in order to drive rapid and accurate protein synthesis. This function requires that EF-Tu must have a sequence and structure that can efficiently interact with GTP, EF-Ts, each of the elongator tRNAs, and the ribosome. EF-Tu has a highly conserved structure which must be capable of undergoing major structural rearrangements during its functional cycle (40, 41). All 17 EF-Tu sequences were aligned with the EF-Tu sequence from *E. coli* (Fig. 4) to aid visualization of amino acid differences between EF-Tus associated with viability and those associated with nonviability. The four viable foreign EF-Tus (green in Fig. 4) show a bias toward a higher conservation of amino acid identity (84% to 94%), relative to *E. coli* EF-Tu, than the 12 nonviable EF-Tus (82% to 69%). It is interesting that there is only a small difference in total percent identity between several of the nonviable EF-Tus (AnEF2, AnEF3, AnEF4, AnEF6, and *B. henselae*, 80 to 82% similarity) and the most distantly related viable EF-Tu (*P. aeruginosa*, 84% similarity), suggesting that the loss of viability in these cases might be associated with changes in a few important residues of EF-Tu.

### Connectivity analysis of EF-Tu reveals an extensive interaction network.

Several studies suggest that protein connectivity in a given network might modulate its activity in the cell (42–44). To examine this, we retrieved an *E. coli* interactome from the HitPredict database (45) and measured the connectivity of EF-Tu in this network. The connectivity of EF-Tu was quantified by measuring its so-called “degree centrality.” Degree centrality is defined as the number of interactions that a given node has in its network. The average degree centrality of all proteins in the *E. coli* interactome is 12, whereas the degree centrality of EF-Tu is much higher at 172. Relative to all proteins in the interactome, EF-Tu ranks among the top 10 most connected proteins in *E. coli* (Table S2). Interestingly, the set of its first interaction partners is enriched in essential proteins (*P* = 3.66 × 10^−21^) (Fig. S4). Accordingly, this analysis suggests the possibility that, in addition to affecting the specific activity of EF-Tu in protein synthesis, additional deleterious effects on fitness might be due to the foreign *tuf* homologs disturbing the extensive interaction network of EF-Tu.

10.1128/mBio.01276-17.4 FIG S4  The interaction partners of EF-Tu and all interactions among these first neighbors of EF-Tu. Nodes represent proteins (indicated by the relevant gene names), and each purple line represents an interaction. To easily distinguish *tufA* in the network, it has been colored cyan. Essential proteins are rectangular and colored blue in this subnetwork. Protein-protein interactions of *E. coli* were retrieved from the HitPredict database (A. Patil, K. Nakai, and H. Nakamura, Nucleic Acids Res 39:D744–D749, 2011, https://doi.org/10.1093/nar/gkq897), and the Cytoscape visualization tool was used to draw the network (B. Demchak et al., F1000Res 3:151, 2014, https://doi.org/10.12688/f1000research.4492.2). Download FIG S4, PDF file, 1 MB. Copyright © 2017 Kacar et al. 2017 Kacar et al. This content is distributed under the terms of the Creative Commons Attribution 4.0 International license.

10.1128/mBio.01276-17.7 TABLE S2  The elongation factor EF-Tu is among the top 10 most connected proteins in the overall *E. coli* interactome. The proteins listed below are ranked according to their degree of connectivity using the UniProt database. The mean average degree of interaction in the whole interactome is 12. Download TABLE S2, PDF file, 0.1 MB. Copyright © 2017 Kacar et al. 2017 Kacar et al. This content is distributed under the terms of the Creative Commons Attribution 4.0 International license.

## DISCUSSION

We generated *E. coli* strains in which the *E. coli tufA* gene was replaced by ancestral and modern homologs of *tuf*, from a broad spectrum of species and ancestral nodes. The origins of the ancestral EF-Tu sequences ranged in age from the Precambrian era, approximately 0.7 bya, back to the last universal common bacterial EF-Tu ancestor, approximately 3.6 bya (Fig. 1). We showed that homologs of EF-Tu encoded by *tuf* genes from within the gammaproteobacteria, including one of the reconstructed ancestral node sequences, AnEF1, are functionally active in *E. coli* and support viability when present as the only *tuf* gene in the chromosome (shown in green in Fig. 2). In contrast, more distantly related homologs and ancestral sequences were unable to support viability as the sole *tuf* gene. Among the four viable homologs, there was a good correlation (*R*^2^ = 0.9371, *P* = 0.032) between phylogenetic distance from *E. coli* EF-Tu and the magnitude of reduced growth fitness for the three homologs from extant species (Fig. 2). The exception was the reconstructed ancestral node sequence, AnEF1, where the decrease in relative fitness was much greater than that predicted by phylogenetic distance (Fig. 2). It could be argued that our definition of viability (ability to construct strains carrying the foreign *tuf* gene and in which the native *tufB* gene was removed) risks excluding some foreign genes because of technical difficulties in constructing the strains. However, the correlation that we observed between reduced relative fitness and reduced amounts of EF-Tu for the viable foreign genes is consistent with the hypothesis that even more distantly related foreign genes might be inviable because they produce an even smaller amount of EF-Tu to support growth. In addition, the observation that many of the “inviable” *tuf* genes were associated with duplication of the *E. coli tufB* region suggests that such strains were under significant selective pressure to compensate for low levels of EF-Tu, consistent with an inadequate level of active EF-Tu associated with the foreign *tuf* gene.

The correlation between phylogenetic distance from *E. coli* and relative growth fitness for the viable homologs raises the question of whether the underlying cause of the reduced fitness is a reduction in the specific activity of the foreign EF-Tus and/or a reduction in the amount of EF-Tu produced. By measuring EF-Tu protein concentration as a function of total protein concentration for each viable strain, including *E. coli* carrying only *tufA*, we observed a strong correlation between relative growth fitness and EF-Tu concentration for each of the four EF-Tus from extant species (Fig. 3). The slope of the correlation suggests that most of the reduced fitness associated with the EF-Tu homologs from the extant species could be attributed to a reduced level of EF-Tu rather than a reduced activity. Consistent with this, previous experiments have shown that reductions in growth rate are associated with reductions in cellular EF-Tu concentrations (46). Once again, the exception to this correlation is found for the reconstructed ancestral AnEF1, where a very high concentration of EF-Tu was associated with a low relative fitness (Fig. 3A), consistent with AnEF1 having a low specific activity. This conclusion is supported by the results of *in vitro* translation, where in a system containing only *T. thermophilus* components (tRNAs, EF-Ts, and ribosomes) AnEF1 supported protein synthesis but with only 30% of the activity of native *E. coli* EF-Tu, demonstrating that AnEF1 can participate in peptide synthesis, albeit in a diminished fashion relative to its modern counterpart EF-Tu (47). To address the significance of the correlation between protein amount and relative fitness, we also overexpressed each of the five viable *tuf* genes (Fig. 3C). We found, in each case where a foreign *tuf* gene was associated with reduced fitness, that the growth rate could be significantly increased by overexpressing the gene. We also found that the step time of protein synthesis varied between the different *tuf* genes and correlated with the relative fitness of each strain. Taken together, these data support the hypothesis that suboptimal expression is a significant cause of the reduced growth fitness associated with the foreign *tuf* genes. However, the data do not rule out the possibility that at least some of the effects are associated with a poor integration of the foreign EF-Tu into the network of interactions that support optimal growth. In principle, reduced fitness associated with a change in specific activity (for example, a weakened interaction) could also be reversed by an increase in the intracellular concentration of EF-Tu. To tease apart the relative contributions to fitness of intracellular EF-Tu concentration from the specific activity of each foreign EF-Tu will require extensive biochemical analysis and is beyond the scope of this paper.

The overexpression of EF-Tu, by recombineering an additional copy at a second chromosomal location, helped answer a question concerning the significance of *tufB* region amplification in many of the strains carrying “inviable” foreign *tuf* genes (Fig. 2). The strains with *tufB* amplified have relatively high fitness values, but their foreign *tuf* genes were subsequently found to be inviable. The question is whether the amplification (which probably includes all genes in a 40-kb region between *rrnB* and *rrnE*) is selected because it increases the copy number of *tuf* genes in the cell or because it increases the copy number of some other gene in the amplified region that increases relative fitness. By constructing strains in which the expression of low-fitness foreign *tuf* genes was individually increased, without increasing the copy number of any other genes, and finding that relative fitness increased as a result, we could conclude that at least in these cases an increase in *tuf* expression was sufficient to increase fitness. It seems reasonable to conclude that the amplification of the region containing *tufB* was most probably also selected because it increased the intracellular concentration of EF-Tu, thus improving fitness.

Why, given that the *tuf* gene regulatory regions are identical in all strains, do the foreign *tuf* coding sequences cause a reduction in the level of EF-Tu produced? Possible explanations include that the nucleotide sequence introduced with the foreign genes affects *tuf* mRNA half-life as previously shown for a mutant of *tuf* (48) or that the altered nucleotide sequence reduces translation efficiency, possibly by effects mediated through altered codon usage or by affecting transcription-translation coupling, as recently shown for *tufB* (30, 49). No correlation was found between differences in the codon usage of the foreign *tuf* genes and the amounts of EF-Tu produced (see Fig. S3 in the supplemental material).

An important question is why some of the not-too-distant foreign homologs are unable to support viability. At the level of total amino acid similarity, there is very little separating viable from nonviable EF-Tu sequences, with the boundary falling at approximately 84% similarity to *E. coli* EF-Tu (Fig. S2). Given the very high level of conservation of EF-Tu, it is possible that the nonviability of some foreign EF-Tus might be related to the alteration of just one or a few critically important residues. Indeed, many single-amino-acid substitutions in EF-Tu have been shown to generate protein variants that do not support viability (50), including one single-amino-acid substitution in EF-Tu that permits ternary complex formation but abolishes translation activity by preventing ternary complex interaction with the ribosome (46). To facilitate an assessment of the amino acid differences between viable and nonviable EF-Tus, their amino acid sequences were aligned (Fig. 4). Of the 393 residues in *E. coli* EF-Tu, there were 116 residues that were identical in all of the viable EF-Tu homologs but differed in at least one of the 12 nonviable homologs. Variation at one or more of these residues might explain the difference between EF-Tu viability and nonviability in *E. coli*. The 116 residues were distributed between each of the three structural domains of EF-Tu, with 48 in the G domain, 38 in domain 2, and 30 in the C-terminal domain 3 (Fig. 4 and S5). Several of the 116 variant residues lie within functionally important regions of EF-Tu, including those involved in coordinating GTP hydrolysis, interaction with EF-Ts, and interaction with the ribosome (51–53). For example, in each of the nonviable EF-Tus there were from 3 to 8 variant residues in regions of the G domain that are known to be functionally important for coordinating the hydrolysis of GTP on EF-Tu during protein synthesis (Fig. S5). These variant residues are located in the conserved regions I and II, the effector region, and the switch I and switch II loops of the G domain of EF-Tu (Fig. S5). The altered residues potentially affecting GTP hydrolysis are at V20, G41, A43, R44, F46, N51, N63, T64, C81, and V88 (Fig. 4). In addition, there are alterations in the P loop, the switch II region, and parts of domain 3 that are involved in interactions with EF-Ts and in helix D of EF-Tu (residues 144 to 156), which is involved in interactions with EF-Ts and protein L7/L12 on the ribosome (52, 53). We can only speculate on the exact reason for the nonviability of these EF-Tu homologs. It seems unlikely that it is directly related to defects in binding or hydrolyzing GTP, given that this process involves highly conserved residues and structures and that the EF-Tus from extant organisms must be capable of supporting viability, including GTP binding and hydrolysis, in their natural system. Similarly, each of the ancestral homologs can support *in vitro* translation, albeit at a low efficiency, arguing that they also can bind and hydrolyze GTP. A similar line of reasoning could also rule out interactions with aa-tRNAs as the cause of nonviability. Perhaps the most plausible reason for nonviability is defective interactions with EF-Ts and/or the ribosome. It seems reasonable to suggest that EF-Tu has coevolved with EF-Ts and the ribosome to modulate the efficiency of these interactions in each species. We suggest accordingly that the cause of nonviability for distantly related EF-Tus is not that they cannot function as enzymes capable of forming a ternary complex and hydrolyzing GTP but rather that they are defective in one or more of the other important interactions made by EF-Tu, namely, with EF-Ts, with the mRNA-programmed ribosome, and possibly even interactions outside protein synthesis involving one or more members of EF-Tu’s extensive protein interaction network (Fig. S4). Coevolution of EF-Tu with its interaction partners would create a barrier to transfer for EF-Tus beyond a certain threshold.

10.1128/mBio.01276-17.5 FIG S5  Structural representation of EF-Tu-GDP. EF-Tu is shaded gray, and residues that are conserved in all viable homologs but changed in at least one of the nonviable homologs are colored. (A) Changes within the G domain are colored red, those in domain II are colored yellow, and those in domain III are colored blue. (B) A close-up view of the G domain where the change in the P loop is shown in green, changes in the effector/switch I region are shown in orange, the change in the switch II region is shown in purple, and changes in the D-helix are shown in blue. GDP is shown in red, and the Mg ion is shown as a cyan sphere. Graphic analyses were performed with the UCSF Chimera package (E. F. Pettersen et al., J Comput Chem 25:1605–1612, 2004, https://doi.org/10.1002/jcc.20084) using the Protein Data Bank identifier 1EFC (H. Song, M. R. Parsons, S. Rowsell, G. Leonard, and S. E. Phillips. J Mol Biol 285:1245–1256, 1999). Download FIG S5, PDF file, 0.8 MB. Copyright © 2017 Kacar et al. 2017 Kacar et al. This content is distributed under the terms of the Creative Commons Attribution 4.0 International license.

We previously hypothesized that evolutionary novelties are more likely to be shared between a descendant and its ancient homolog than between two currently existing protein homologs (54). Accordingly, replacing an existing gene with its ancient homolog may have a smaller negative fitness impact on the organism relative to exchanging the native gene with a currently existing homolog. However, functional divergence occurring through time could result in ancestral sequences being so maladapted to the new host cell that a functional organism is all but precluded (55). This limitation does not apply only to ancestral genes, and it has been suggested that as the number of edges connecting a protein within its protein-protein interaction network increases, the probability that a protein could be successfully replaced with a homolog will decrease even if there is a functional equivalence between the endogenous gene and the homolog (2). While a careful assessment of candidate ancestral protein properties prior to integration is helpful, in most cases, studying gene-triggered genomic perturbations experimentally through the integration of ancestral genes offers a valuable and complementary alternative to existing methodologies that use extant homologous proteins (56–59).

How can we identify the specific historical constraints on replacement? We observe that only the ancient EF-Tu representing an ancestor within the gammaproteobacteria, AnEF1 (0.7 bya), and the modern EF-Tu homologs from extant gammaproteobacteria are viable. In contrast, the last common ancestor of the alpha-, beta-, and gammaproteobacteria, AnEF6 (1.3 bya), is nonviable. Accordingly, we speculate that mutational substitutions in EF-Tu occurring between 1.6 bya and 0.7 bya influenced the replaceability of *tuf* genes. These mutations may constrain *tuf* replaceability by disturbing EF-Tu’s functional interaction with other cellular components, ultimately impacting its participation in protein synthesis. Thus, extensive mutational remodeling of interaction partners may be necessary in order to engineer even older ancient *tuf* genes inside the bacteria.

### Conclusions.

We show that foreign *tuf* genes encoding EF-Tu proteins exhibit suboptimal functionality and reduced fitness when introduced into another host. The suboptimality of the foreign *tuf* genes most likely results from disturbances in interactions directly important for protein synthesis, but suboptimal EF-Tu protein levels and disturbance of other potentially important interactions in the network of EF-Tu might also play a role. The observation that the only *tuf* homologs that supported viability belong to the gammaproteobacterial taxon, or an associated ancestral node within the gammaproteobacteria, suggests that there is a relatively stringent “transferability cutoff,” i.e., a point in the phylogeny beyond which functional divergence is too great for replacement. For EF-Tu protein, this transferability zone is within the ancestral and modern gammaproteobacterial taxon, unlike some ribosomal proteins where constraints on replaceability are less stringent (22, 60).

Future efforts may involve identifying protein sites that interfere with organismal-level function and epistatically inhibit an ancient protein’s function in a descendant organism. Our experiments suggest that a protein like EF-Tu, which is highly conserved and involved in multiple highly conserved interactions, is so highly optimized and finely tuned in the host organism that it is essentially irreplaceable by distantly related foreign genes. The degree to which epistatic interactions constrain EF-Tu replaceability and functionality in the cell needs to be studied more to deepen our understanding of the design principles of complex biological systems and to allow us to introduce alterations in modern organisms by genetic engineering and gene replacements.

## MATERIALS AND METHODS

### Media and growth conditions.

In general, liquid and solid media used were Luria-Bertani (LB) medium (per liter, 10 g NaCl [5 g in the case of low-salt LB], 5 g yeast extract, and 10 g tryptone) and LA (LB with 1.5% agar) plates. Where indicated, growth in minimal medium was made in Davis minimal (DM) medium [per liter, 7 g K_2_HPO_4_, 2 g KH_2_PO_4_, 1 g (NH_4_)_2_SO_4_, 0.5 g sodium citrate]. For *sacB* counterselections, the LB and LA media used had no NaCl, and LA medium was also supplemented with 5% sucrose. All incubations were made at 37°C (unless stated otherwise), and liquid cultures were shaken at 200 rpm for aeration. The antibiotics used (Sigma-Aldrich, Sweden) had the following final concentrations: 50 mg/liter kanamycin and 15 mg/liter tetracycline.

### Growth parameter measurements.

To measure doubling time, lag time, and maximum OD at 600 nm (OD_600_) of the strains in LB and DM25 (DM with 25 g/liter glucose), 3 independent cultures of each strain were grown overnight in LB. For each culture, 2 aliquots of 400 µl were washed with NaCl_2_ to remove any traces of the medium and then resuspended in 400 µl of LB or DM25, respectively. Subsequently, 280 µl of a 100-fold dilution of every aliquot was added to 3 wells of the microtiter honeycomb plate, as technical replicates. Control wells were filled with 280 µl of only LB or DM25. Bacterial growth was monitored by measuring the rate of increase in optical density at OD_600_ using a Bioscreen C machine (Oy Growth Curves Ab Ltd., Finland) and growing the cultures in the honeycomb microtiter plate. Plates were incubated for 24 h at 37°C with continuous shaking, and readings of OD_600_ were taken at 5-min intervals. All data points were corrected by subtracting the OD_600_ of the corresponding control wells (medium only) at every time point and then converted to log values. The doubling time (DT) of each strain during exponential growth was calculated over an interval of 50 min (10 time points) in the linear region of the curve by calculating the slope of the interval using the following equation:

(1) $$\text{DT}=\frac{\text{ln}\left(2\right)}{\text{slope}}$$

Lag times were estimated over the same time interval using the following equation:

(2) $$\text{lag time}=\frac{\text{initial OD}\;-\;y\;\text{intercept}}{\text{slope}}$$

Maximum OD_600_ was defined as the maximum optical density over 24 h of monitored growth. Relative fitness, defined in terms of doubling time in LB, was calculated by comparing each independent doubling time measurement to the average of doubling time measurements of the strain carrying *E. coli tufA*.

### Bacterial strains.

All strains used in this study are derived from sequenced wild-type *E. coli* K-12 strain MG1655 (61), unless stated otherwise. A list of the strains used in these experiments is shown in Table S3 in the supplemental material.

10.1128/mBio.01276-17.8 TABLE S3  Strains and genetic markers used in this work and their origin. Download TABLE S3, PDF file, 0.1 MB. Copyright © 2017 Kacar et al. 2017 Kacar et al. This content is distributed under the terms of the Creative Commons Attribution 4.0 International license.

### Strain construction.

The genetic marker (TP22-amilCP_opt-kan-sacB-T0) was inserted into the chromosome to replace either *tufA* or *tufB* by double-stranded DNA lambda-red recombineering (62, 63). The lambda-red genes were induced in each strain from the temperature-sensitive pSIM5-tet plasmid by incubation of an overday culture (OD_600_ of 0.3) at 43°C for 15 min. After cooling for 10 min in ice, the cells were made electrocompetent by washing in ice-cold water three times. Electroporation of TP22-amilCP_opt-kan-sacB-T0 PCR product was done with a Gene Pulser (Bio-Rad, USA) by mixing 50 µl of electrocompetent cells and 100 ng of the cassette, with settings of 1.8 kV, 25 µF, and 200 Ω. Cells were recovered in 1 ml of low-salt LB at 30°C overnight with agitation, and after recovery, 100 µl of the culture was spread in LA plates containing kanamycin for selection of recombinants.

Genetic markers were moved between strains by phage-mediated (P1 *virA*) transduction. Lysates of the strains carrying the marker were made by mixing 1 ml of overnight culture containing 5 mM CaCl_2_ with 100 µl of the P1 virA lysate previously made on *E. coli* MG1655. The bacterium-phage mix was incubated for 10 min, and then 4 ml of soft agar (LB medium plus 0.8% agar plus 5 mM CaCl_2_) was added; this mixture was spread over an LA plate and incubated overnight. To release the bacteriophages, the soft agar was mixed with 4 ml and vortexed. The resultant slurry was centrifuged for 15 min at 5,000 rpm, and the supernatant was filtered through an 0.2-µm filter. Markers were transduced into the desired recipient strain by mixing 100 µl of the lysate with 500 µl of an overnight culture containing 5 mM CaCl_2_. After 10 min of incubation, 100 µl of the mixture was spread onto a selective plate.

Insertions of alien *tuf* genes were made by double-stranded DNA lambda-red recombineering to replace the previously inserted counterselectable TP22-amilCP_opt-kan-sacB-T0 marker. The accession numbers and/or references for the foreign *tuf* genes are given in Table S4 in the supplemental material. To make clean deletions, markers were deleted by single-stranded lambda-red recombineering with counterselection for sucrose resistance (64), following the same steps as described above for the double-stranded DNA lambda-red recombineering.

10.1128/mBio.01276-17.9 TABLE S4  List of organisms and plasmids serving as EF-Tu sources. Download TABLE S4, PDF file, 0.1 MB. Copyright © 2017 Kacar et al. 2017 Kacar et al. This content is distributed under the terms of the Creative Commons Attribution 4.0 International license.

To construct the strains expressing an extra copy of the *tuf*, gene, two promoters of different strengths (J23105 and J23100) (iGEM Registry of Standard Biological Parts, Cambridge, MA; http://partsregistry.org) transcriptionally fused to a *cat-sacB* cassette at the *galK* locus were transduced into strains carrying the relevant single *tuf* gene. Coding sequences for the *tuf* genes were amplified from each of the strains carrying a single *tuf* gene and engineered by recombineering behind each of the two promoters replacing the *cat-sacB* cassette. Constructs were confirmed by PCR and DNA sequencing (Macrogen Europe Laboratory, Amsterdam, The Netherlands).

### Ancestral gene reconstruction.

Ancestral sequences used in this study originated from the study performed by Gaucher et al. (35). Briefly, the EF-Tu sequences were retrieved from GenBank database, and the phylogenetic tree was constructed with MrBayes (65). Ancestral sequences were calculated with PAML (66).

### PCR and oligonucleotides.

PCR was performed on an S1000 Thermal Cycler (Bio-Rad, USA). Oligonucleotides were designed with the software CLC Main workbench 7 (CLC bio, Denmark) using the genome of *E. coli* MG1655 as reference. For generation of the TP22-amilCP_opt-kan-sacB-T0 cassette, PCR was performed using Phusion High-Fidelity PCR master mix with HF buffer (New England Biolabs, USA) and with the following cycling conditions: 98°C for 30 s and 30 cycles of 98°C for 10 s, 55°C for 30 s, 72°C for 4 min, and 72°C for 7 min. For routine diagnostic PCR, Fermentas PCR master mix (Thermo Scientific, USA) was used with the following cycling conditions: 95°C for 5 min and 30 cycles of 95°C for 30 s, annealing temperature (*T*_*A*_) for 30 s, 72°C for elongation time (*E*_*T*_), and 72°C for 5 min. *T*_*A*_ varied depending on the pair of primers used, and *E*_*T*_ was based on the length of the expected product (30 s per kilobase). Oligonucleotides for construction of strains, PCR, and sequencing are shown in Table S5.

10.1128/mBio.01276-17.10 TABLE S5  Oligonucleotide primers used in this study. Download TABLE S5, PDF file, 0.1 MB. Copyright © 2017 Kacar et al. 2017 Kacar et al. This content is distributed under the terms of the Creative Commons Attribution 4.0 International license.

### Preparation of genomic DNA and real-time quantitative PCR.

Genomic DNA prepared using the MasterPure DNA purification kit (Epicentre Biotechnologies, USA) was used to run real-time quantitative PCR. One microliter genomic DNA (gDNA) (diluted 1:10, 1:100, 1:1,000, and 1:10,000), 10 μl PerfeCTa SYBR green FastMix (Quanta Biosciences), 0.6 μl of 10 μM forward and reverse primers, and double-distilled water (ddH_2_O) were added to a final reaction volume of 20 μl. The Eco real-time PCR system (Illumina) was used for running the PCR. Oligonucleotides amplifying the *rpoB* and *purD* genes were used to quantify the amplification status of the *rrnB-rrnE* region. The *cysG* and *indT* genes were used as controls. The oligonucleotide sequences used as RT-qPCR primers are listed in Table S5.

### Whole-genome sequencing and analysis.

Genomic DNA was prepared using the MasterPure DNA purification kit (Epicentre Biotechnologies, USA). To create libraries of paired-end fragments, the Nextera XT sample preparation kit (Illumina, USA) was used according to the instructions from the manufacturer. Sequencing was performed on the Illumina MiSeq instrument, generating 250-bp paired-end reads. Whole-genome sequencing data were analyzed using the CLC Genomic Workbench software (CLC bio, Denmark).

### Local DNA sequencing.

Local sequencing of PCR-amplified products was performed at the Macrogen Europe sequencing facilities (Amsterdam, The Netherlands), and data were analyzed using the CLC Main Workbench 7 software (CLC bio, Denmark).

### Protein synthesis rate measurements (step time).

Liquid cultures were initiated from overnight cultures grown in LB to mid-log phase at 37°C with shaking by diluting 1:100 in 20 ml DM, with 0.2% glycerol. Before induction, a time zero sample (200 μl) was taken and added to 300 μl ice-cold chloramphenicol (0.5 mg/ml in 1:1 H_2_O-ethanol). Expression of *lacZ* from the F′23 plasmid was induced by the addition of 200 ml IPTG (isopropyl-β-d-thiogalactopyranoside; 0.1 M; final concentration, 1 mM). Samples (200 μl) were taken every 20 s after induction for 300 s and added to 300 μl chloramphenicol solution. Cells were pelleted by centrifugation (3 min, 12,000 × *g*) and resuspended in 300 μl Z buffer (0.06 M Na_2_HPO_4_⋅2H_2_O, 0.04 M NaH_2_PO_4_⋅H_2_O, 0.1 M KCl, 0.001 M MgSO_4_⋅7H_2_O, 0.05 M β-mercaptoethanol). To each sample, 100 μl chloroform and 50 μl 0.1% SDS were added. The tubes were vortexed and left on ice for 20 min to allow the chloroform to sink before 200 μl of each sample was added to a honeycomb plate with 40 μl *o*-nitrophenyl-β-d-thiogalactopyranoside (ONPG; 4 mg/ml) added per well. The plate was incubated with shaking in a Bioscreen C machine (Oy Growth Curves AB Ltd.), and absorbance at 420 nm and 540 nm was measured. Background absorbance (ONPG in Z buffer without cells) and absorbance at time zero were subtracted, and the data were plotted with √[(OD_420_) − (1.75 × OD_540_)] as a function of time. The intercept with the *x* axis of the induced curve is the step time, the time that it takes to produce the first β-galactosidase activity. To calculate the protein synthesis rate in amino acids/second, the length of the β-galactosidase (1,024 amino acids) is divided by the step time of each strain. The relative protein synthesis rate was determined in each case by comparing the protein synthesis rate to the average of all measurements of the strain carrying *E. coli tuf*.

### Statistical analysis.

All statistical analyses were performed using GraphPad Prism v6.0c (GraphPad Software, Inc., USA). The significance of differences between fitness costs was calculated using an unpaired two-tailed *t* test.

### Proteomics: cell lysis, digestion, and labeling procedures.

All cells were placed into Covaris microTUBE-15 (Woburn, MA) microtubes with Covaris TPP buffer. Samples were lysed in a Covaris S220 focused ultrasonicator instrument with 125-W power over 180 s with 10% maximum peak power. Lysed cells were digested via filter-aided sample preparation (FASP) digest according to the FASP protocol for trypsin digestion, followed by high-pressure liquid chromatography (HPLC) purification. We used Promega sequencing-grade trypsin-LysC (V5073; Madison, WI) overnight at 38°C. Each sample was submitted for a single LC-MS/MS experiment that was performed on an LTQ Orbitrap Elite (Thermo Fisher) equipped with a Waters (Milford, MA) NanoAcquity HPLC pump or Orbitrap Lumos (Thermo Fisher, San Jose, CA) equipped with EasyLC1000 (Thermo Fisher, San Jose, CA). Peptides were separated onto a 100-µm-inner-diameter microcapillary trapping column packed first with approximately 5 cm of C_18_ Reprosil resin (5 µm, 100 Å; Dr. Maisch GmbH, Germany), followed by an analytical column with ~20 cm of Reprosil resin (1.8 µm, 200 Å; Dr. Maisch GmbH, Germany). Separation was achieved by applying a gradient from 5 to 27% acetonitrile (ACN) in 0.1% formic acid over 90 min at 200 nl min^−1^. Electrospray ionization was enabled by applying a voltage of 1.8 kV using a homemade electrode junction at the end of the microcapillary column and spraying from fused silica pico tips (New Objective, MA). The LTQ Orbitrap Elite/Lumos was operated in data-dependent mode for the mass spectrometry methods. The mass spectrometry survey scan was performed in the Orbitrap in the range of 395 to 1,800 *m/z* at a resolution of 6 × 10^4^, followed by the selection of the 20 most intense ions (TOP20) for collision-induced dissociation (CID)–MS2 fragmentation in the ion trap using a precursor isolation width window of 2 *m/z*, an AGC (automatic gain control) setting of 10,000, and a maximum ion accumulation of 200 ms. Singly charged ion species were not subjected to CID fragmentation. Normalized collision energy was set to 35 V and an activation time of 10 ms. Ions in a 10-ppm *m/z* window around ions selected for MS2 were excluded from further selection for fragmentation for 60 s. The same TOP20 ions were subjected to an HCD (Higher-energy collisional dissociation) MS2 event in the Orbitrap part of the instrument. The fragment ion isolation width was set to 0.7 *m/z*, the AGC was set to 50,000, the maximum ion time was 200 ms, normalized collision energy was set to 27 V, and an activation time of 1 ms for each HCD MS2 scan was used.

### Mass spectrometry analysis.

Raw data were submitted for analysis in Proteome Discoverer 2.1.0.81 (Thermo Scientific) software. Assignment of MS/MS spectra was performed using the Sequest HT algorithm by searching the data against a protein sequence database including all entries from the user database and our *E. coli* K-12 database as well as other known contaminants such as human keratins and common lab contaminants. Sequest HT searches were performed using a 20-ppm precursor ion tolerance and requiring each peptide’s N/C termini to adhere with trypsin protease specificity, while allowing up to two missed cleavages. Six-plex tandem mass tags (TMTs) on peptide N termini and lysine residues (+229.162932 Da) were set as static modifications, while methionine oxidation (+15.99492 Da) was set as a variable modification. An MS2 spectrum assignment false discovery rate (FDR) of 1% on the protein level was achieved by applying the target-decoy database search. Filtering was performed using a 64-bit Percolator. For quantification, an 0.02 *m/z* window was centered on the theoretical *m/z* value of each of the six reporter ions and the intensity of the signal closest to the theoretical *m/z* value was recorded. Reporter ion intensities were exported in a result file of the Proteome Discoverer 2.1 search engine as an Excel table.

### Evolutionary divergence.

Two independent approaches were utilized in order to estimate the evolutionary distance between sequences, both leading to the same evolutionary distance estimate output. (i) For MEGA software, the analyses involved 18 sequences. Analyses were conducted using the Poisson correction model (67). All positions containing gaps and missing data were eliminated. There were a total of 385 positions in the final data set. Evolutionary analyses were conducted in MEGA7 (68). (ii) The branch length distances were also calculated via a custom script that used ETE software (69). The custom Python ETE v.3 Python library script is provided in the supplemental material.

### Computational methods.

Computational analyses include (i) the pairwise and multiple sequence alignments, (ii) protein structure analysis, and (iii) protein interaction network analysis. Clustal Omega (70) was used to perform multiple sequence alignment with default parameters. Pairwise sequence alignments of the wild-type EF-Tu against each ancient and modern EF-Tu protein were performed by EMBOSS Needle of Clustal Omega with default parameters, which uses the Needleman-Wunsch alignment algorithm.

The protein interactome of *E. coli* was retrieved from the HitPredict (45) database, and the network analysis was performed with the network package of Python (71). The subnetwork of EF-Tu interaction partners was visualized with Cytoscape (72).
