# Supplementary figures and images for: Functional Constraints on Replacing an Essential Gene with Its Ancient and Modern Homologs
Source: mBio. 2017 Aug 29;8(4):e01276-17. doi: 10.1128/mBio.01276-17 (PMC5574714; doi:10.1128/mBio.01276-17)

**A**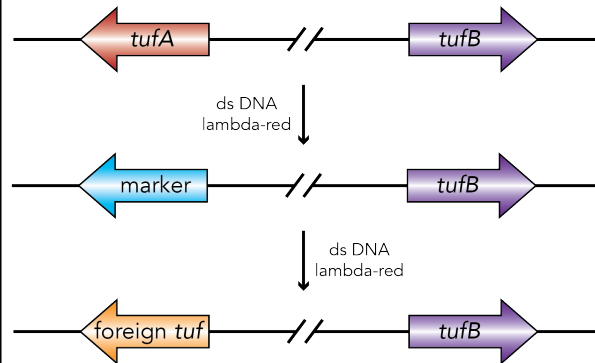**B**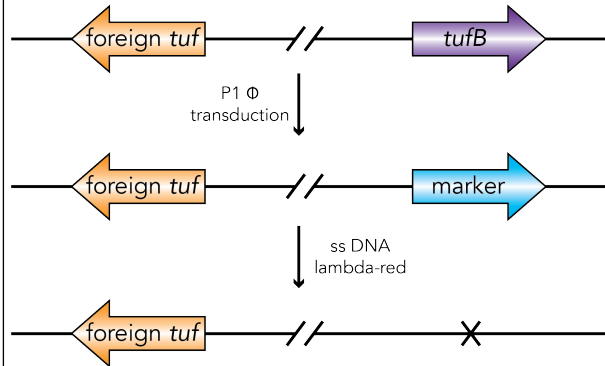

Supplement: FIG S1 [file mbo004173450sf1.pdf]

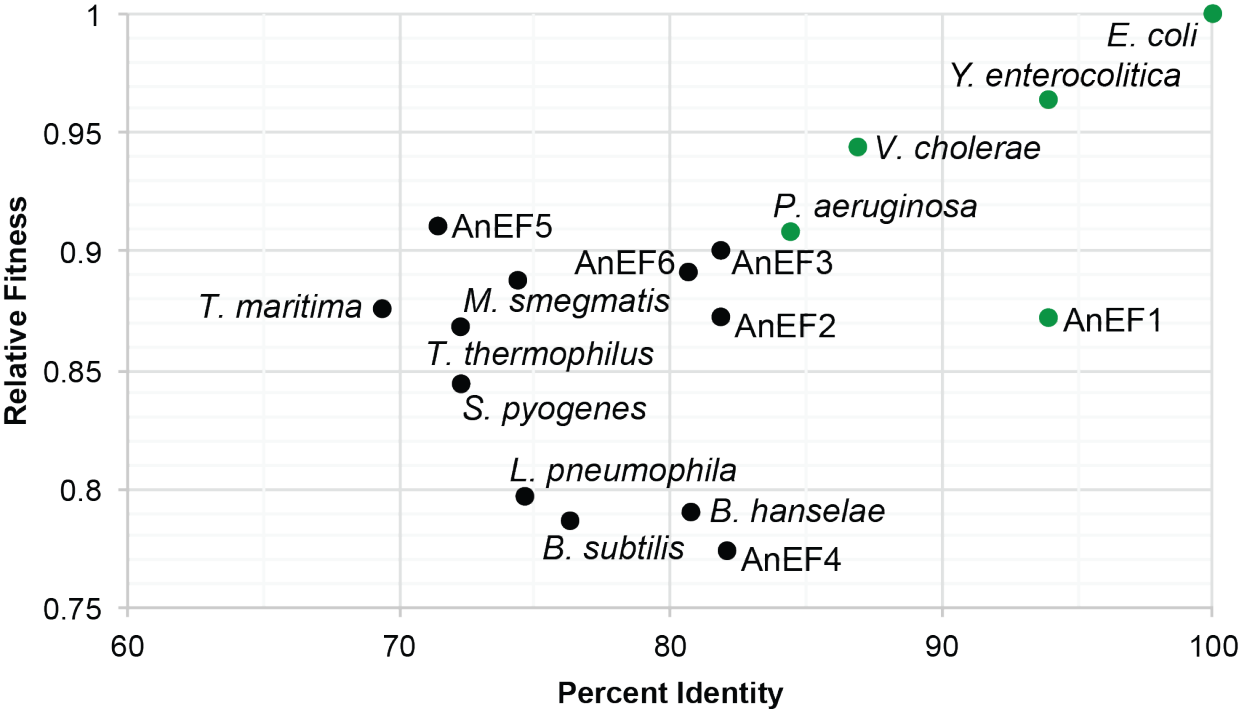

Supplement: FIG S2 [file mbo004173450sf2.pdf]

**A**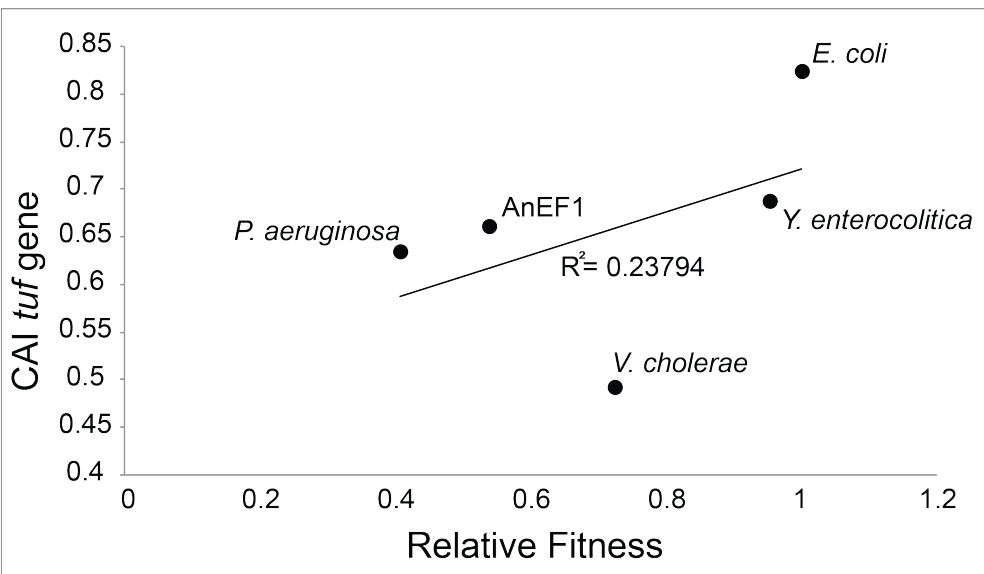**B**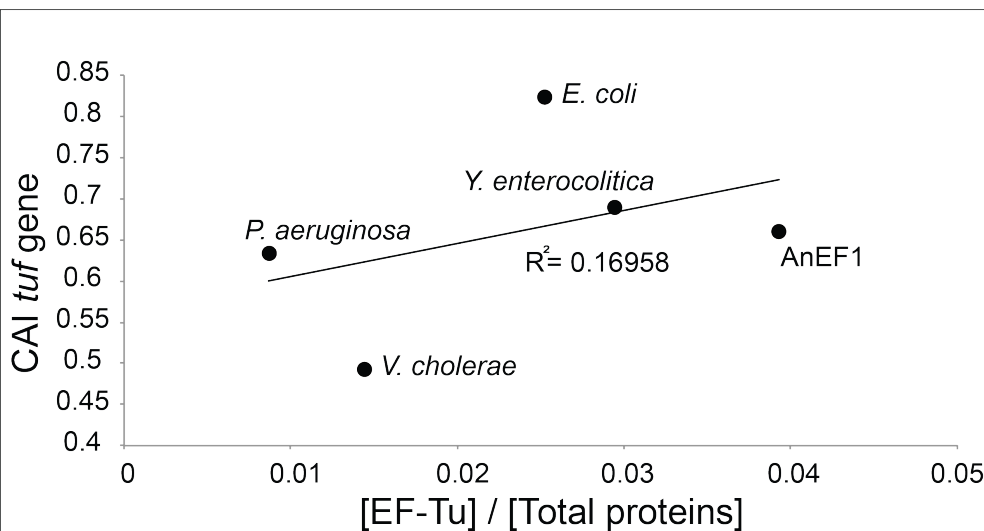

Supplement: FIG S3 [file mbo004173450sf3.pdf]

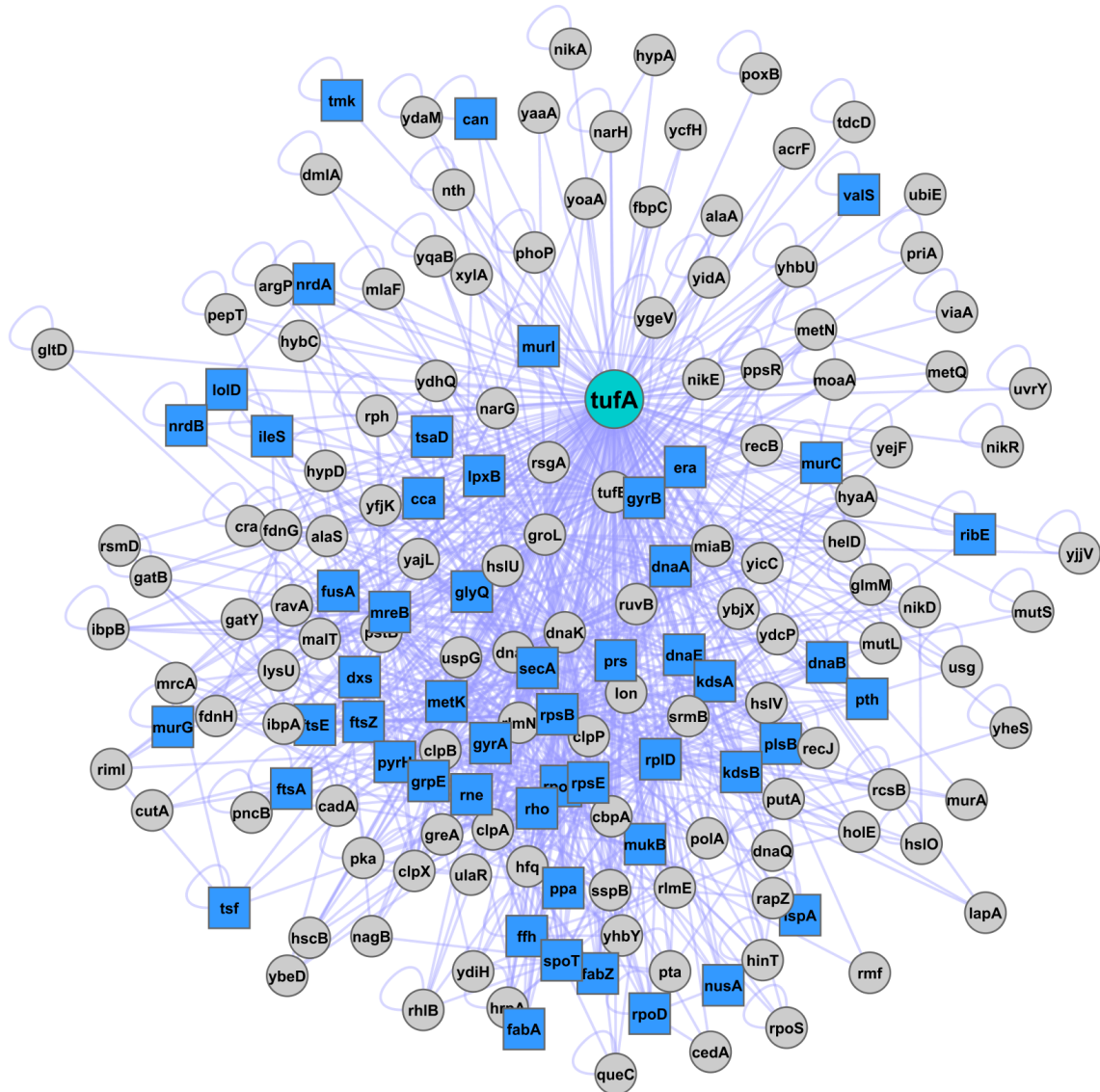

Supplement: FIG S4 [file mbo004173450sf4.pdf]

A

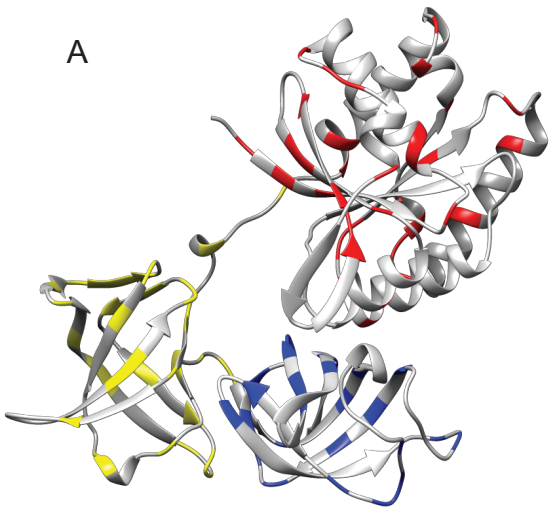

B

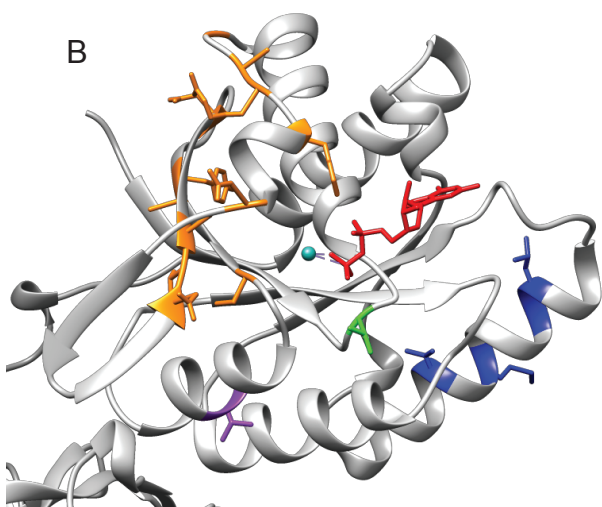

Supplement: FIG S5 [file mbo004173450sf5.pdf]
