# Supplementary material for: Functional Constraints on Replacing an Essential Gene with Its Ancient and Modern Homologs
Source: mBio. 2017 Aug 29;8(4):e01276-17. doi: 10.1128/mBio.01276-17 (PMC5574714; doi:10.1128/mBio.01276-17)
Supplement: TABLE S1 [file mbo004173450st1.pdf]

Table S1. Nucleotide sequences of ancestral *tuf* genes

Ancestral gene	Sequence
AnEF1	GTGTCCAAAGAGAAATTTGAACGCACGAAACCACATGTAAACGTAGGTACCATTGGC<br>CACGTTGATCATGGCAAAACTACCCTGACTGCGGCAATTACCACGGTACTGGCGAAA<br>ACTTACGGCGGTGCAGCTCGTGCTTTCGACCAGATCGATAACGCTCCGGAGGAGAAA<br>GCTCGTGGCATCACCATCAATACGTCCCACGTTGAATACGATACGCCTACCCGTCAT<br>TACGCTCATGTAGATTGTCCGGGCCACGCGGACTATGTTAAGAACATGATCACCGGC<br>GCAGCTCAGATGGACGGTGCAATCCTGGTTGTGGCTGCTACCGATGGCCCGATGCCG<br>CAGACGCGTGAACACATTCTGCTGGGCCGTCAGGTTGGCGTACCGTACATTATCGTG<br>TTTCTGAACAAATGCGACATGGTCGACGATGAAGAACTGCTGGAGCTGGTCGAAATG<br>GAGGTGCGCGAACTGCTGTCCCAGTACGACTTCCCGGGCGATGATACCCCGATCATC<br>CGTGGTTCTGCTCTGAAGGCTCTGGAAGGCGAAGCAGAATGGGAGGCGAAAATTATC<br>GAGCTGGCCGAAGCGCTGGACAGCTACATCCCAGAACCGGAACGTGCAATCGATCAG<br>CCGTTTCTGCTGCCGATTGAAGATGTTTTCAGCATCTCCGGTCGTGGTACGGTAGTT<br>ACCGGCCGTGTGGAACGTGGCATCGTAAAAGTTGGTGAAGAAGTCGAAATTGTTGGT<br>ATCAAAGATACTACCAAAACTACCTGCACCGGCGTGGAAATGTTCCGCAAACTGCTG<br>GACGAAGGCCGTGCGGGCGAAAACGTAGGTGTCCTGCTGCGCGGCACCAAACGCGAC<br>GAAATCGAGCGTGGTCAGGTTCTGGCGAAACCGGGCTCCATCACCCCTCACACGACT<br>TTTGAATCCGAGGTGTACGTGCTGTCTAAAGACGAAGGTGGCCGTCACACTCCATTC<br>TTCAAGGGCTATCGTCCGCAGTTCTACTTTCGCACCACGGACGTGACCGGTACCATT<br>GAACTGCCTGAAGGTGTTGAGATGGTTATGCCGGGCGACAACATTAAAATGACCGTG<br>ACTCTGATCCACCCTATTGCGATGGACGAAGGTCTGCGTTTTGCCATCCGTGAAGGT<br>GGTCGTACCGTTGGCGCCGGTGTGGTTGCGAAAATCATCGCTTAA
AnEF2	GTGGCCAAAGAGAAATTTGAACGCACGAAACCACATGTAAACATCGGTACCATTGGC<br>CACGTTGATCATGGCAAAACTACCCTGACTGCGGCAATTACCAAGGTACTGGCGGAG<br>AAAGGCCAAGCTGAATTTAAGGCTTACGACCAGATCGATAAGGCTCCGGAGGAGAAA<br>GAACGTGGCATCACCATCAGCACGGCCCACGTTGAATACGAGACTGAAAACCGTCAT<br>TACGCTCATGTAGATTGTCCGGGCCACGCGGACTATGTTAAGAACATGATCACCGGC<br>GCAGCTCAGATGGACGGTGCAATCCTGGTTGTGTCTGCTGCCGATGGCCCGATGCCG<br>CAGACGCGTGAACACATTCTGCTGGCCCGTCAGGTTGGCGTACCGTACATTGTCGTG<br>TTTCTGAACAAAGTCGACATGGTCGACGATGAAGAACTGCTGGAGCTGGTCGAAATG<br>GAGGTGCGCGAACTGCTGTCCTCTTACGACTTCCCGGGCGATGATATCCCGATCATC<br>AAAGGTTCTGCTCTGAAGGCTCTGGAAGGCGACGAAGAAGGCGAGGAGGCAATTATG<br>AAGCTGATGGACGCGGTGGACAGCTACATCCCAGAACCGGAACGTGCAATCGATAAG<br>CCGTTTCTGATGCCGATTGAAGATGTTTTCAGCATCTCCGGTCGTGGTACGGTAGTT<br>ACCGGCCGTGTGGAACGTGGCATCGTAAAAGTTGGTGAAGAAGTCGAAATTGTTGGT<br>ATCCGTGATACTCAGAAAACTACCTGCACCGGCGTGGAAATGTTCCGCAAACTGCTG<br>GACGAAGGCCAAGCGGGCGACAACGTAGGTGTCCTGCTGCGCGGCACGAAACGCGAA<br>GATGTCGAGCGTGGTCAGGTTCTGGCCAAACCGGGCTCCATCAAACCTCACACGAAA<br>TTTAAAGCCGAGGTGTACATCCTGACTAAAGAAGAAGGTGGCCGTCACACTCCATTC<br>TTCAACGGCTATCGTCCGCAGTTCTACTTTCGCACCACGGACGTGACCGGTGTGATT<br>ACCCTGCCTGAAGGTGTTGAGATGGTTATGCCGGGCGACAACGTTACTATCACCGTG<br>GAACTGATCGCCCCTATTGCGATGGAAGAAGGTCTGCGTTTTGCCATTCGTGAAGGT<br>GGTCGTACCGTTGGCGCCGGTGTGGTTTCCGAAATCATCGAATAA
AnEF3	GTGGCCAAAGAGAAATTTGAACGCACGAAACCACATGTAAACATCGGTACCATTGGC<br>CACGTTGATCATGGCAAAACTACCCTGACTGCGGCAATTACCAAGGTTCTGGCGGAG<br>AAAGGCAAAGCTGAATTTCGTGCTTACGACCAGATCGATAAGGCTCCGGAGGAGAAA<br>GAGCGTGGCATCACCATCAACACGGCCCACGTTGAATACGAGACTGAAAACCGTCAT

	TACGCTCATGTAGATTGTCCGGGCCACGCGGACTATGTTAAGAACATGATCACCGGC<br>GCAGCTCAGATGGACGGTGCAATCCTGGTTGTGTCTGCTGCCGATGGCCCGATGCCG<br>CAGACGCGTGAACACATTCTGCTGGCCCGTCAGGTTGGCGTACCGTACATTGTCGTG<br>TTTCTGAACAAATGCGACATGGTCGACGATGAAGAACTGCTGGAGCTGGTCGAAATG<br>GAGGTGCGCGAACTGCTGTCCGAGTACGACTTCCCGGGCGATGATATCCCGATCATC<br>CGCGGTTCTGCTCTGAAGGCTCTGGAAGGCGATGAAGAATGGGTGGAAAAGATTATG<br>GAGCTGATGGACGCGGTGGACAGCTACATCCCAGAACCGGAACGTGACATCGATAAG<br>CCGTTTCTGATGCCGATTGAAGATGTTTTCAGCATCACCGGTCGTGGTACGGTAGTT<br>ACCGGCCGTGTGGAACGTGGCATCGTAAAAGTTGGTGACGAAGTCGAAATTGTTGGT<br>CTGCGTGATACTCGTAAAACTGTCGTCACCGGCGTGGAAATGTTCCGCAAACTGCTG<br>GACGAAGGCCAGGCGGGCGACAACGTAGGTGTCCTGCTGCGCGGCATCAAACGCGAA<br>GATGTCGAGCGTGGTCAGGTTCTGGCCAAACCGGGCTCCATCAAACCTCACACGAAA<br>TTTAAAGCCGAGGTGTACGTGCTGACTAAAGAAGAAGGTGGCCGTCACACTCCATTC<br>TTCAACGGCTATCGTCCGCAGTTCTACTTTCGCACCACGGACGTGACCGGTGTGATT<br>ACCCTGCCTGAAGGTGTTGAGATGGTTATGCCGGGCGACAACGTTACTATGACCGTG<br>GAACTGATCCACCCTATTGCGATGGAAGAAGGTCTGCGTTTTGCCATCCGTGAAGGT<br>GGTCGTACCGTTGGCGCCGGTGTGGTTTCCGAAATCATCGAATAA
AnEF4	GTGGCCAAAGAGAAATTTGTACGCACGAAACCACATGTAAACGTCGGTACCATTGGC<br>CACGTTGATCATGGCAAATCTACCCTGACTGCGGCAATTACCAAGTATCTGTCTCTG<br>AAAGGCCTGGCTCAATATGTTCCTTACGACCAGATCGATAAGGCTCCGGAGGAGAAA<br>GCTCGTGGCATCACCATCAACATTACCCACGTTGAATACGAGACTGAGAAACGTCAT<br>TACGCTCATATCGATTGTCCGGGCCACGCGGACTATATTAAGAACATGATCACCGGC<br>GCAGCTCAGATGGACGGTGCAATCCTGGTTGTGGCTGCTACCGATGGCCCGATGCCG<br>CAGACGCGTGAACACGTTCTGCTGGCCCGTCAGGTTGGCGTACCGTACATGATCGTG<br>TTTATCAACAAAACCGACATGGTCGACGATCCAGAACTGATCGAGCTGGTCGAAATG<br>GAGGTGCGCGATCTGCTGTCCCAGTACGAATATCCGGGCGATGAAGTCCCGGTCATC<br>AAGGGTTCTGCTCTGAAGGCTCTGGAAGCCAACCATGAAGCGTATAAACCAATTCAG<br>GAGCTGCTGGACGCGATGGACAACTACATCCCAGACCCGCAACGTGACGTCGATAAG<br>CCGTTTCTGATGCCGATTGAAGATGTTTTCAGCATCACCGGTCGTGGTACGGTAGTT<br>ACCGGCCGTATCGAACGTGGCCGCATCCGTCCTGGTGACGAAGTCGAAATTATTGGT<br>CTGTCTGAGATTCGTAAAACTGTCGTCACCAGCGTGGAAATGTTCCGCAAAGAGCTG<br>GACGAAGGCATTGCGGGCGACAACGTAGGTTGCCTGCTGCGCGGCATCGACAAAGAT<br>GAAGTCGAGCGTGGTCAGGTTCTGGCCGCACCGGGCTCCATCAAACCTCACAAGCGT<br>TTTAAAGCCGAGGTGTACGTGCTGAAGAAAGAAGAAGGTGGCCGTCACACTCCATTC<br>TTCAAAGGCTATAAACCGCAGTTCTACATTCGCACCACGGACGTGACCGGTGAGATT<br>GTCCTGCCTGAAGGTGTTGAGATGGTTATGCCGGGCGACCACGTTGAAATGGAAATC<br>GAACTGATCTACCCTGTTGCGATTGAAAAGGGTCAGCGTTTTGCCATCCGTGAAGGT<br>GGTCGTACCGTTGGCGCCGGTGTGGTTACCGAAGTCATCGAATAA
AnEF5	GTGGCCAAAGAGAAATTTGAACGCACGAAACCACATGTAAACATCGGTACCATTGGC<br>CACGTTGATCATGGCAAAACTACCCTGACTGCGGCAATTACCAAGACTCTGGCGGCG<br>AAAGGCAAAGCTGAAGCTCGTGCTTACGACCAGATCGATAAGGCTCCGGAGGAGAAA<br>GCTCGTGGCATCACCATCAACACGGCCCACGTTGAATACGAGACTGAAAACCGTCAT<br>TACGCTCATGTAGATTGTCCGGGCCACGCGGACTATGTTAAGAACATGATCACCGGC<br>GCAGCTCAGATGGACGGTGCAATCCTGGTTGTGTCTGCTGCCGATGGCCCGATGCCG<br>CAGACGCGTGAACACATTCTGCTGGCCCGTCAGGTTGGCGTACCGTACATTGTCGTG<br>TTTCTGAACAAATGCGACATGGTCGACGATGAAGAACTGCTGGAGCTGGTCGAAATG<br>GAGGTGCGCGAACTGCTGTCCGAGTACGAATTTCCGGGCGATGATATCCCGATCATC<br>CGCGGTTCTGCTCTGAAGGCTCTGGAAGGCGAAAATGAATGGGTGGACAAAATTTGG<br>GAGCTGATGGACGCGGTGGACAGCTACATCCCAACCCCGGAACGTGACGTCGATAAG

	CCGTTTCTGATGCCGGTTGAAGATGTTTTCACCATCACCGGTCGTGGTACGGTAGCT<br>ACCGGCCGTGTGGAACGTGGCACCGTAAAAGTTGGTGACGAAGTCGAAATTGTTGGT<br>CTGCGTGATACTCGTAAAACTGTCGTCACCGGCGTGGAAATGTTCCGCAAACTGCTG<br>GACGAAGGCATGGCGGGCGACAACGTAGGTGTCCTGCTGCGCGGCATCCAACGCGAA<br>GATGTCGAGCGTGGTCAGGTTCTGGCCAAACCGGGCTCCATCAAACCTCACACGAAA<br>TTTGAAGCCGAGGTGTACGTGCTGACTAAAGAAGAAGGTGGCCGTCACACTCCATTC<br>TTCAACGGCTATCGTCCGCAGTTCTACTTTCGCACCACGGACGTGACCGGTGTGATT<br>ACCCTGCCTGAAGGTGTTGAGATGGTTATGCCGGGCGACAACGTTACTATGACCGTG<br>GAACTGATCCACCCTATTGCGATGGAAGAAGGTCTGCGTTTTGCCATCCGTGAAGGT<br>GGTCGTACCGTTGGCGCCGGTGTGGTTTCCAAAATCATCGAATAA
AnEF6	GTGGCCAAAGAGAAATTTGAACGCACGAAACCACATGTAAACATCGGTACCATTGGC<br>CACGTTGATCATGGCAAAACTACCCTGACTGCGGCAATTACCAAGACTCTGGCGGCG<br>AAAGGCAAAGCTGAAGCTCGTGCTTACGACCAGATCGATAAGGCTCCGGAGGAGAAA<br>GCTCGTGGCATCACCATCAACACGGCCCACGTTGAATACGAGACTGAAAACCGTCAT<br>TACGCTCATGTAGATTGTCCGGGCCACGCGGACTATGTTAAGAACATGATCACCGGC<br>GCAGCTCAGATGGACGGTGCAATCCTGGTTGTGTCTGCTGCCGATGGCCCGATGCCG<br>CAGACGCGTGAACACATTCTGCTGGCCCGTCAGGTTGGCGTACCGTACATTGTCGTG<br>TTTCTGAACAAATGCGACATGGTCGACGATGAAGAACTGCTGGAGCTGGTCGAAATG<br>GAGGTGCGCGAACTGCTGTCCGAGTACGAATTTCCGGGCGATGATATCCCGATCATC<br>CGCGGTTCTGCTCTGAAGGCTCTGGAAGGCGAAAATGAATGGGTGGACAAAATTTGG<br>GAGCTGATGGACGCGGTGGACAGCTACATCCCAACCCCGGAACGTGACGTCGATAAG<br>CCGTTTCTGATGCCGGTTGAAGATGTTTTCACCATCACCGGTCGTGGTACGGTAGCT<br>ACCGGCCGTGTGGAACGTGGCACCGTAAAAGTTGGTGACGAAGTCGAAATTGTTGGT<br>CTGCGTGATACTCGTAAAACTGTCGTCACCGGCGTGGAAATGTTCCGCAAACTGCTG<br>GACGAAGGCATGGCGGGCGACAACGTAGGTGTCCTGCTGCGCGGCATCCAACGCGAA<br>GATGTCGAGCGTGGTCAGGTTCTGGCCAAACCGGGCTCCATCAAACCTCACACGAAA<br>TTTGAAGCCGAGGTGTACGTGCTGACTAAAGAAGAAGGTGGCCGTCACACTCCATTC<br>TTCAACGGCTATCGTCCGCAGTTCTACTTTCGCACCACGGACGTGACCGGTGTGATT<br>ACCCTGCCTGAAGGTGTTGAGATGGTTATGCCGGGCGACAACGTTACTATGACCGTG<br>GAACTGATCCACCCTATTGCGATGGAAGAAGGTCTGCGTTTTGCCATCCGTGAAGGT<br>GGTCGTACCGTTGGCGCCGGTGTGGTTTCCAAAATCATCGAATAA
