# Supplementary material for: Functional Constraints on Replacing an Essential Gene with Its Ancient and Modern Homologs
Source: mBio. 2017 Aug 29;8(4):e01276-17. doi: 10.1128/mBio.01276-17 (PMC5574714; doi:10.1128/mBio.01276-17)
Supplement: TABLE S2 [file mbo004173450st2.pdf]

Table S2: The elongation factor EF-Tu is among the top ten most connected proteins in the overall *E. coli* interactome. The proteins listed below are ranked according to their degree of connectivity using the Uniprot database. The mean average degree of interaction in the whole interactome is 12.

Rank	Uniprot Code	Gene	Product	Connectivity
1	P0A6F5	*groL*	GroEL, chaperone Hsp60, peptide-dependent ATPase	702
2	P18843	*nadE*	NH(3)-dependent NAD(+) synthetase	483
3	P60422	*rplB*	50S ribosomal protein L2	386
4	P0A6Y8	*dnaK*	Chaperone protein DnaK	363
5	P60723	*rplD*	50S ribosomal protein L4	244
6	P0A7V8	*rpsD*	30S ribosomal protein S4	242
7	P0A7V3	*rpsC*	30S ribosomal protein S3	225
8	P0A7V0	*rpsB*	30S ribosomal protein S2	206
9	P0A7W1	*rpsE*	30S ribosomal protein S5	192
10	P0CE47	*tufA*	Elongation factor TuA	172
