# Supplementary material for: Functional Constraints on Replacing an Essential Gene with Its Ancient and Modern Homologs
Source: mBio. 2017 Aug 29;8(4):e01276-17. doi: 10.1128/mBio.01276-17 (PMC5574714; doi:10.1128/mBio.01276-17)
Supplement: TABLE S3 [file mbo004173450st3.pdf]

Supplementary Table 3: Strains and genetic markers used in this work and their origin.

Strain	Species, Genotype or Characteristics
CH1464	*Escherichia coli* K12 MG1655
CH3139	*Pseudomonas aeruginosa* PAO1
CH3998	MG1655 *yfaH*::[TP22-amilCP_opt-kan-*sacB*-T0]
CH1940	MG1655 / pSIM5-tet
CH4765	MG1655 *tufA*::[TP22-amilCP_*opt-kan-sacB*-T0]
CH4766	MG1655 *tufB*::[TP22-amilCP_*opt-kan-sacB*-T0]
CH4849	MG1655 *tufA*::[TP22-amilCP_*opt-kan-sacB*-T0] / pSIM5-tet
CH4932	*Bacillus subtilis*
CH4935	*Streptococcus pyogenes*
CH4961	MG1655 *tufA*::*tuf* from *Mycobacterium smegmatis*
CH4962	MG1655 *tufA*::*tuf* from *Streptococcus pyogenes*
CH4963	MG1655 *tufA*::*tuf* from *Thermus thermophilus*
CH4964	MG1655 *tufA*::*tufB* from *Vibrio cholerae*
CH4965	MG1655 *tufA*::*tuf* from *Bacillus subtilis*
CH4966	MG1655 *tufA*::*tuf* from *Thermotoga maritima*
CH4967	MG1655 *tufA*::AnEF4
CH4968	MG1655 *tufA*::AnEF5
CH4969	MG1655 *tufA*::AnEF6
CH4970	MG1655 *tufA*::AnEF1
CH4971	MG1655 *tufA*::AnEF2
CH4972	MG1655 *tufA*::AnEF3
CH5018	MG1655 *tufB*::[TP22-amilCP_*opt-kan-sacB*-T0], tufA::tuf from *Mycobacterium smegmatis*
CH5019	MG1655 *tufB*::[TP22-amilCP_*opt-kan-sacB*-T0], *tufA*::*tuf* from *Streptococcus pyogenes*
CH5020	MG1655 *tufB*::[TP22-amilCP_*opt-kan-sacB*-T0], *tufA*::*tuf* from *Thermus thermophilus*
CH5021	MG1655 *tufB*::[TP22-amilCP_*opt-kan-sacB*-T0], *tufA*::*tufB* from *Vibrio cholerae*
CH5022	MG1655 *tufB*::[TP22-amilCP_*opt-kan-sacB*-T0], *tufA*::*tuf* from *Bacillus subtilis*

CH5023	MG1655 *tufB*::[TP22-amilCP_*opt-kan-sacB-T0*], *tufA*::*tuf* from *Termotoga maritima*
CH5024	MG1655 *tufB*::[TP22-amilCP_*opt-kan-sacB*-T0], *tufA*::AnEF4
CH5025	MG1655 *tufB*::[TP22-amilCP_*opt-kan-sacB*-T0], *tufA*::AnEF5
CH5026	MG1655 *tufB*::[TP22-amilCP_*opt-kan-sacB*-T0], *tufA*::AnEF6
CH5027	MG1655 *tufB*::[TP22-amilCP_*opt-kan-sacB*-T0], *tufA*::AnEF1
CH5028	MG1655 *tufB*::[TP22-amilCP_*opt-kan-sacB*-T0], *tufA*::AnEF2
CH5029	MG1655 *tufB*::[TP22-amilCP_*opt-kan-sacB*-T0], *tufA*::AnEF3
CH5089	MG1655 *tufB*::[TP22-amilCP_*opt-kan-sacB*-T0], *tufA*::*tufA* from *Vibrio cholerae* / pSIM5-tet
CH5091	MG1655 Δ*tufA*
CH5130	MG1655 *tufB*::[TP22-amilCP_*opt-kan-sacB*-T0], *tufA*::AnEF1 / pSIM5-tet
CH5133	MG1655 Δ*tufB*, *tufA*::*tufA* from *Vibrio cholerae*
CH5226	MG1655 *tufB*::[TP22-amilCP_*opt-kan-sacB*-T0] / pSIM5-tet
CH5227	MG1655 *tufA*::*tufB* from *Legionella pneumophila* Philadelphia 1
CH5228	MG1655 *tufA*::*tuf2* from *Bartonella hanselae* Houston 1
CH5229	MG1655 *tufB*::*tuf* from *Bacillus subtilis*
CH5234	MG1655 Δ*tufB*
CH5235	MG1655 Δ*tufB*, *tufA*::AnEF1
CH5239	*Yersinia enterocolitica*
CH5240	MG1655 *tufB*::[TP22-amilCP_*opt-kan-sacB*-T0], *tufA*::*tufB* from *Legionella pneumophila* Philadelphia 1
CH5241	MG1655 *tufB*::[TP22-amilCP_*opt-kan-sacB*-T0], *tufA*::*tuf2* from *Bartonella henselae* Houston 1
CH5244	MG1655 *tufA*::*tufA* from *Yersinia enterocolitica*
CH5249	MG1655 *tufA*::*tufA* from *Pseudomonas aeruginosa*
CH5256	MG1655 *tufB*::[TP22-amilCP_*opt-kan-sacB*-T0], tufA::tufA from Yersinia enterocolitica
CH5258	MG1655 *tufB*::[TP22-amilCP_*opt-kan-sacB*-T0], *tufA*::*tufA* from *Pseudomonas aeruginosa*
CH5269	MG1655 *tufB*::[TP22-amilCP_*opt-kan-sacB*-T0], *tufA*::*tufA* from *Yersinia enterocolitica* / pSIM5-tet
CH5271	MG1655 *tufB*::[TP22-amilCP_*opt-kan-sacB*-T0], *tufA*::*tufA* from *Pseudomonas aeruginosa* / pSIM5-tet
CH5277	MG1655 Δ*tufB*, *tufA*::*tufA* from *Yersinia enterocolitica*
CH5279	MG1655 Δ*tufB*, *tufA*::*tufA* from *Pseudomonas aeruginosa*

CH7238	MG1655 Δ*tufB*, Δ*lac-pro* F'23 lac+ proAB+
CH7239	MG1655 Δ*tufB*, *tufA*::*tufA* from *Vibrio cholerae*, Δ*lac-pro* F'23 lac+ proAB+
CH7240	MG1655 Δ*tufB*, *tufA*::*tufA* from *Yersinia enterocolitica*, Δ*lac-pro* F'23 lac+ proAB+
CH7241	MG1655 Δ*tufB*, *tufA*::*tufA* from *Pseudomonas aeruginosa*, Δ*lac-pro* F'23 lac+ proAB+
CH7242	MG1655 Δ*tufB*, *tufA*::AnEF1, Δ*lac-pro* F'23 lac+ proAB+
CH7453	MG1655 Δ*tufB*, *tufA*::*tufA* from *Vibrio cholerae*, J23100-*tufA(V.cholerae*)<>*galK*
CH7454	MG1655 Δ*tufB*, *tufA*::*tufA* from *Yersinia enterocolitica*, J23100-*tufA*(*Y.enterocolitica*)<>*galK*
CH7455	MG1655 Δ*tufB*, *tufA*::*tufA* from *Pseudomonas aeruginosa*, J23100-*tufA*(*P.aeruginosa*)<>*galK*
CH7456	MG1655 Δ*tufB*, *tufA*::AnEF1, J23100-AnEF1<>*galK*
CH7457	MG1655 Δ*tufB*, J23105-tufA(*E.coli*)<>*galK*
CH7458	MG1655 Δ*tufB*, *tufA*::*tufA* from *Vibrio cholerae*, J23105-*tufA*(*V.cholerae*)<>*galK*
CH7459	MG1655 Δ*tufB*, *tufA*::*tufA* from *Yersinia enterocolitica*, J23105-*tufA*(*Y.enterocolitica*)<>*galK*
CH7460	MG1655 Δ*tufB*, *tufA*::*tufA* from *Pseudomonas aeruginosa*, J23105-*tufA*(*P.aeruginosa*)<>*galK*
CH7461	MG1655 Δ*tufB*, *tufA*::AnEF1, J23105-AnEF1<>*galK*
