# Supplementary material for: Functional Constraints on Replacing an Essential Gene with Its Ancient and Modern Homologs
Source: mBio. 2017 Aug 29;8(4):e01276-17. doi: 10.1128/mBio.01276-17 (PMC5574714; doi:10.1128/mBio.01276-17)
Supplement: TABLE S4 [file mbo004173450st4.pdf]

Table S4. List of organisms and plasmids serving as EF-Tu sources.

Organism/node	Strain	Accession number	Annotation
Vibrio cholerae	N16961 El Tor	NC_016445	*tufB*
Mycobacterium smegmatis	MC2 155	NC_018289	*tuf*
Bacillus subtilis	186	NC_000964	*tuf*
Thermus thermophilus	HB8	ATCC 27634	*tuf*
Thermotoga maritima	DSM3109 [MSB8]	ATCC 43589	*tuf*
Streptococcus pyogenes	MGAS5005	NC_007297	*tuf*
Legionella pneumophila	Philadelphia 1	AE017354	*tufB*
Bartonella hanselae	Houston 1	BX897699	*tuf2*
Yersinia enterocolitica	8081	NC_008800	*tufA*
Pseudomonas aeruginosa	PAO1	NC_002516	*tufA*
Node 317 (700mya)	Synthetic gene	N/A	AnEF1
Node 253 (~1.2bya)			AnEF2
Node (~1.8bya)			AnEF3
Node 168 (~2.5bya)			AnEF4
Node 184 (~3.5bya)			AnEF5
Node 262 (~2.5bya)			AnEF6
