# Supplementary material for: Functional Constraints on Replacing an Essential Gene with Its Ancient and Modern Homologs
Source: mBio. 2017 Aug 29;8(4):e01276-17. doi: 10.1128/mBio.01276-17 (PMC5574714; doi:10.1128/mBio.01276-17)
Supplement: TABLE S5 [file mbo004173450st5.pdf]

Table S5. Oligonucleotide primers used in this study

Oligo name	Oligo sequence (5' to 3')
tufB::kansaccolor_fw	GTGATATCACCGATTTATCCGTGTCTTAGAGGGACAATCGATCAAAGGGAAAACTGTCCA
tufB::kansaccolor_rv	ATGCCCTTTTAGTGCGCATTGCGTCAAATGTTATCGGCAAAAAATGAGACGTTGATCGGCACG
tufB_coli_fw	TTCTTTTCTCCTCCCTGT
tufB_coli_rv	GGCAAACCAAATCGAAAC
tufA::topo1.317_fw	CCGAAGCGCCCTTTTCAATTCAAAACTAATTAACGTGTAATTAAGCGATGATTTTCGCAACCA
tufA::topo1.317_rv	CTCTCCTGAAGGGGAGAGCACTATAGTAAGGAATATAGCCGTGTCCAAAGAGAAATTTGAACG
tufA::topo2.262_fw	CCGAAGCGCCCTTTTCAATTCAAAACTAATTAACGTGTAATTATTCGATGATTTTGGAAACCA
tufA::topo2.262_rv	CTCTCCTGAAGGGGAGAGCACTATAGTAAGGAATATAGCCGTGGCCAAAGAGAAATTTGAACG
tufA::topo2.253_fw	CCGAAGCGCCCTTTTCAATTCAAAACTAATTAACGTGTAATTATTCGATGATTTCGGAAACCA
tufA::topo2.253_rv	CTCTCCTGAAGGGGAGAGCACTATAGTAAGGAATATAGCCGTGGCCAAAGAGAAATTTGAACG
tufA::topo1.170_fw	CCGAAGCGCCCTTTTCAATTCAAAACTAATTAACGTGTAATTATTCGATGATTTCGGAAACCA
tufA::topo1.170_rv	CTCTCCTGAAGGGGAGAGCACTATAGTAAGGAATATAGCCGTGGCCAAAGAGAAATTTGAACG
tufA::topo1.168_fw	CCGAAGCGCCCTTTTCAATTCAAAACTAATTAACGTGTAATTATTCGATGACTTCGGTAACCA
tufA::topo1.168_rv	CTCTCCTGAAGGGGAGAGCACTATAGTAAGGAATATAGCCGTGGCCAAAGAGAAATTTGTACG
tufA::topo2.184_fw	CCGAAGCGCCCTTTTCAATTCAAAACTAATTAACGTGTAATTATTCGATGATTTTGGAAACCA
tufA::topo2.184_rv	CTCTCCTGAAGGGGAGAGCACTATAGTAAGGAATATAGCCGTGGCCAAAGAGAAATTTGAACG
tufA::tufA.sty_fw	CCGAAGCGCCCTTTTCAATTCAAAACTAATTAACGTGTAATTAGCCCAGAACTTTAGCAA
tufA::tufA.sty_rv	CTCTCCTGAAGGGGAGAGCACTATAGTAAGGAATATAGCCGTGTCTAAAGAAAAATTTGA
tufA::tufB.vcholerae_fw	CCGAAGCGCCCTTTTCAATTCAAAACTAATTAACGTGTAATTAAGCGATGATCTTAGCTACAA
tufA::tufB.vcholerae_rv	CTCTCCTGAAGGGGAGAGCACTATAGTAAGGAATATAGCCGTGTCTAAAGAGAAATTTGAACG
tufA::tuf.msmeg_fw	CCGAAGCGCCCTTTTCAATTCAAAACTAATTAACGTGTAATTACTTGATGATCTTGGTGACGC
tufA::tuf.msmeg_rv	CTCTCCTGAAGGGGAGAGCACTATAGTAAGGAATATAGCCGTGGCGAAGGCGAAGTTCGAGCG
tufA::tuf.bsub_fw	CGAAGCGCCCTTTTCAATTCAAAACTAATTAACGTGTAATTACTCAGTGATTGTAGAAACAAC
tufA::tuf.bsub_rv	CTCTCCTGAAGGGGAGAGCACTATAGTAAGGAATATAGCCGTGGCTAAAGAAAAATTCGACCG
tufA::tuf.thermophilus_fw	CCGAAGCGCCCTTTTCAATTCAAAACTAATTAACGTGTAATTATTCCAGGATTTTGGTAACCA
tufA::tuf.thermophilus_rv	CTCTCCTGAAGGGGAGAGCACTATAGTAAGGAATATAGCCGTGGCCAAAGGTGAATTTATCCG
tufA::tuf.maritima_fw	CCGAAGCGCCCTTTTCAATTCAAAACTAATTAACGTGTAATTATTCGATGACTTCGGTAACCA
tufA::tuf.maritima_rv	CTCTCCTGAAGGGGAGAGCACTATAGTAAGGAATATAGCCGTGGCCAAAGAGAAATTTGTACG
tufA::tuf.spyogenes_fw	CCGAAGCGCCCTTTTCAATTCAAAACTAATTAACGTGTAATTAAGCTTCGATTTCTGAAACGA
tufA::tuf.spyogenes_rv	CTCTCCTGAAGGGGAGAGCACTATAGTAAGGAATATAGCCGTGGCAAAAGAAAAATACGATCG

del_kansacblue_tufB_coli	CTTTTAGTGCGCATTGCGTCAAATGTTATCGGCAACGATTGTCCCTCTAAGACACGGATAAATCGGTGAT
del_kansacblue_tufA_coli	GCGCCCTTTTCAATTCAAAACTAATTAACGTGTAAGGCTATATTCCTTACTATAGTGCTCTCCCCTTCAG
tufs_check_fw	CAAAACAAAAGTCCTACGCA
tufs_check_rv	AGTATGATGAAGCGCCGA
check_deltufA_coli_fw	GTCAACAAATGCAAAAAGGG
check_deltufA_coli_rv	AAACTCCGGAAGAGAACAC
check_deltufB_coli_fw	TCCCCCACCACCAATTTC
check_deltufB_coli_rv	CCCACTTCATCGCTTCCA
tufA::tuf.legionella_fw	CCGAAGCGCCCTTTTCAATTCAAAACTAATTAACGTGTAATTACTCGATTATTTTAGCGA
tufA::tuf.legionella_rv	TCTCTCCTGAAGGGGAGAGCACTATAGTAAGGAATATAGCCGTGGCGAAGGAAAAATTTGAACG
tufA::tuf.bartonella_fw	CCGAAGCGCCCTTTTCAATTCAAAACTAATTAACGTGTAATTACTCAATGATCTTAGAAA
tufA::tuf.bartonella_rv	CTCTCCTGAAGGGGAGAGCACTATAGTAAGGAATATAGCCGTGGCGAAGAGCAAATTTGAACG
tufA::tuf.yersinia_fw	CCGAAGCGCCCTTTTCAATTCAAAACTAATTAACGTGTAATTAAGCGATAACTTTAGCAA
tufA::tuf.yersinia_rv	CTCTCCTGAAGGGGAGAGCACTATAGTAAGGAATATAGCCGTGTCTAAAGAAAAATTTGAACG
tufA_yersinia_fw	TTGAATGGTATGTGGGTG
tufA_yersinia_rv	TGGAGTTCCTGAAGTATG
tufA::tuf.pseudomonas_fw	CCGAAGCGCCCTTTTCAATTCAAAACTAATTAACGTGTAATTACTCGATGATCTTGGCAA
tufA::tuf.pseudomonas_rv	ATGCCCTTTTAGTGCGCATTGCGTCAAATGTTATCGGCAATTACTCGATGATCTTGGCAA
tufA_pseudomonas_fw	CCGGATTTGTTGGTTTTG
tufA_pseudomonas_rv	TGGGTGAGATGTTCGGTT
rpoB_qPCR_fw	TAAGGTAACGCCGAAAGGTG
rpoB_qPCR_rv	CAGAGGCTTTCTCACCGAAG
purD_qPCR_fw	GCGAGCTTTATCGTGATGGT
purD_qPCR_rv	CCTACGCGTTTGTGATCCTG
cysG_qPCR_fw	TTGTCGGCGGTGGTGATGTC
cysG_qPCR_rv	ATGCGGTGAACTGTGGAATAAACG
hcaT_qPCR_fw	GCTGCTCGGCTTTCTCATCC
hcaT_qPCR_rv	CCAACCACGCTGACCAACC
